# Functional annotation of uncharacterized proteins from *Fusobacterium nucleatum*: identification of virulence factors

**DOI:** 10.5808/gi.22065

**Published:** 2023-06-30

**Authors:** Kanchan Rauthan, Saranya Joshi, Lokesh Kumar, Divya Goel, Sudhir Kumar

**Affiliations:** Department of Biotechnology, H.N.B. Garhwal University, Srinagar Garhwal, Uttarakhnd 246174, India

**Keywords:** *Fusobacterium nucleatum*, functional annotation, hypothetical proteins, uncharacterized proteins, virulence factors

## Abstract

*Fusobacterium nucleatum* is a gram-negative bacteria associated with diverse infections like appendicitis and colorectal cancer. It mainly attacks the epithelial cells in the oral cavity and throat of the infected individual. It has a single circular genome of 2.7 Mb. Many proteins in *F. nucleatum* genome are listed as “Uncharacterized.” Annotation of these proteins is crucial for obtaining new facts about the pathogen and deciphering the gene regulation, functions, and pathways along with discovery of novel target proteins. In the light of new genomic information, an armoury of bioinformatic tools were used for predicting the physicochemical parameters, domain and motif search, pattern search, and localization of the uncharacterized proteins. The programs such as receiver operating characteristics determine the efficacy of the databases that have been employed for prediction of different parameters at 83.6%. Functions were successfully assigned to 46 uncharacterized proteins which included enzymes, transporter proteins, membrane proteins, binding proteins, etc. Apart from the function prediction, the proteins were also subjected to string analysis to reveal the interacting partners. The annotated proteins were also put through homology-based structure prediction and modeling using Swiss PDB and Phyre2 servers. Two probable virulent factors were also identified which could be investigated further for potential drug-related studies. The assigning of functions to uncharacterized proteins has shown that some of these proteins are important for cell survival inside the host and can act as effective drug targets.

## Introduction

*Fusobacterium nucleatum* strain ATCC 25586 is an anaerobic, gram-negative, and opportunistic pathogen which belongs to Bacteroidaceae family. *Fusobacterium* mostly inhabits the oral cavity and throat of the diseased as well as normal individuals by adhering (through FadA protein) and invading the epithelial cells of mouth and gut. This bacterium forms a biofilm and alters the host immune response through the process of adhesion and invasion of critical organs like head, neck, lung, liver, heart, and brain. *Fusobacterium* infection can cause diseases like periodontitis, gingivitis, and appendicitis [1].

It has been reported that *Fusobacterium* crosses the placenta and causes preterm and still birth in women having pregnancy-associated gingivitis [2]. This bacterium is also found to be associated with colorectal cancer progression in those patients who have been suffering from long-term intestinal bowel disease [3]. Antibiotics therapy and surgical treatment (in some cases) are used to treat several diseases caused by *F. nucleatum*. Continuous administration of these antibiotics can cause antimicrobial resistance in bacteria. Therefore, the identification of new therapeutic target and development of new drugs against this bacterium can help in reducing the burden of disease.

Shotgun genome sequencing of *F. nucleatum* strain ATCC 25586 reveals 2.17 Mb of genome containing a single circular chromosome having 27% GC content and 2,067 open reading frames [4]. Some of these open reading frames have been listed as “hypothetical” or “uncharacterized” proteins. These hypothetical proteins (HPs) are functionally and structurally uncharacterized and are classified into uncharacterized protein families and domain of unknown function classes. While many proteins are characterized during the sequencing itself, some of them remain uncharacterized due to lack of better sequence homolog or structurally related protein. It is therefore important to revisit such uncharacterized proteins and assign them functions in the context of new scientific knowledge. These previously uncharacterized proteins may yield interesting results and shed some light on functionality of a cell [5]. Many researchers have used this computer guided approach to functionally annotate the uncharacterized protein or HP from different organisms [6-8]. In the present study, we have attempted the functional annotation of uncharacterized proteins present in the genome of *F. nucleatum* strain ATCC 25586. Out of 398 uncharacterized proteins listed in *F. nucleatum* genome, we have assigned functions to 39 sequences with high confidence and another 7 with relatively low confidence. The receiver operating characteristics (ROC) analysis performed to evaluate the methodology adopted, yielded an average accuracy of 83 % across the parameters.

## Methods

### Sequence retrieval

Proteome data of *F. nucleatum* strain ATCC 25586 was downloaded using Proteome ID UP000002521 format from UniProt database. Proteome of *F. nucleatum* strain ATCC 25586 contains 2,046 proteins, out of which 398 proteins are listed as ‘uncharacterized proteins’. FASTA sequences of these 398 uncharacterized proteins were used for further analysis.

### Physicochemical properties

Physical and chemical properties of a protein such as molecular weight, extinction coefficient, isoelectric point, grand average of hydropathicity, etc. were estimated through Expasy’s ProtParam program [9]. ProtParam computes physicochemical properties using the protein sequence only. Negative grand average value of hydropathicity (GRAVY) shows the hydrophilic nature of protein and vice-versa. The instability index of less than 40 indicates a stable protein.

### Sub-cellular localization

Prediction of protein localization was done through Cello server. It uses a hybrid approach i.e., support vector machines model and a structural homology approach for localization prediction [10]. SignalP 5.0 was used for predicting signal peptide and cleavage site in a protein’s sequence. Signal peptide is a small sequence present on the protein which directs them for movement to target position in the cell. Signal peptides are generally cleaved by signal peptidases after the translocation. SignalP 5.0 uses a deep neural network-based approach to identify the signal peptides [11]. TMHMM server was used for predicting the presence of transmembrane helices in a protein [12].

### Domain identification

The protein sequences were then subjected to domain identification using the InterProScan, Motif, Smart, HMMER, NCBI CDART (Conserved Domain Architecture Retrieval Tool), and BlastP programs.

InterProScan server classifies the protein sequences into homologous superfamily and identifies the functional domain based on information compiled from different databases. Besides this, InterProScan can also identify the presence of signal peptide and transmembrane helices in protein sequences [13]. Motif server was used for identification of motifs in a protein sequence which is available in GenomeNet database. SMART (Simple Modular Architecture Research Tool) web server in combination with UniProt, Ensembl, and String database analyze the domain architecture using per-species protein clustering procedure (normal mode) and completely sequenced genome (genomic mode) [14]. HMMER web server uses jack-hmmer algorithm for the annotation of protein sequence based on identified domain [15]. NCBI CDART performs an RPS-BLAST against Entrez protein database for domain similarity in query protein sequence [16]. BlastP uses heuristic approach to identify the sequence similarity between input sequences and database sequences [17].

### Performance assessment

ROC, a web-based calculator was used to check the accuracy, sensitivity, and specificity of the different servers used in this study (Supplementary Table 1) [18]. About 50 proteins with known functions were randomly selected from *F. nucleatum* (Supplementary Table 1) and their functions were predicted against the same databases as used for the prediction of HPs in this study. Six-level classification of predictions were done using binary (0,1) format in which 0 represents true negative and 1 represents true positive. Integers ‘2’, ‘3’, ‘4’, and ‘5’ were used for the diagnosis of efficacy, higher the integer, higher the efficacy. ROC web server generates a ROC curve between sensitivity and 1-specificity, where area under the curve represents the effective measures of accuracy ranging from 0 to 1. The average accuracy and ROC area of the used database/s were determined to be 83.6% and 0.90, respectively (Supplementary Table 2).

### String analysis

The functional partners reveal important information about a protein and its function. To search for such information, we subjected the 46 annotated proteins to the string database search [19]. After preliminary analysis, proteins with a confidence score of >1 were listed.

### Homology detection with the human proteins

All the 46 annotated proteins were searched in BLASTp program against the human proteins (taxon id: 9606) in non-redundant database. These proteins were also searched in DrugBank database for identification of any similar druggable candidates [20].

### Structure prediction and modeling

Homology-based structural modelling was carried out for the annotated proteins using Swiss PDB [21] and Phyre2 servers [22]. Templates with most sequence coverage were subsequently used for model building. Structure models were predicted for 25 annotated proteins with identity ranging from 14% to 97%. Based on the annotations, we identified several proteins which might play important role in cell survival and therefore, can be a potential drug target. Models of some of these proteins were explored further. The structure models were uploaded on PDBSum [23] page and their structural quality was assessed by PROCHECK [24].

### Virulence prediction

The annotated protein sequences were analyzed for virulence factor prediction using the VICMPred [25] and VirulentPred [26] softwares. Proteins which were determined as virulent factors by both programs were analyzed further.

**Fig. 1.**
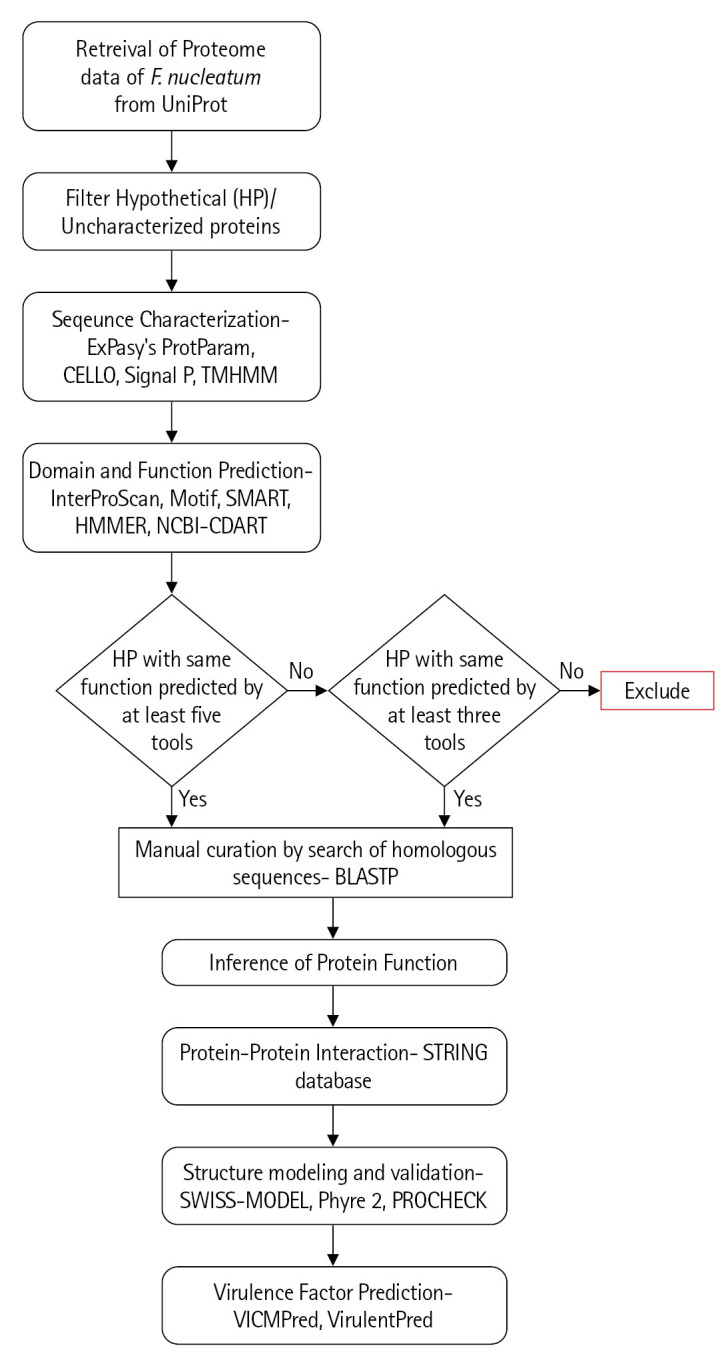
Flowchart of methodology. The methodology adopted for functional annotation of uncharacterized proteins of *Fusobacterium nucleatum* included the servers for sequential characterization, sub-cellular location, domain and motif identification, structure prediction, and virulence factor identification. Only the results with high confidence are taken and the rest are excluded.

The characterization of the previously uncharacterized proteins employed a large number of available programs and servers which predict the essential parameters such as localization, domains, motifs, interactions, etc. The inference of the probable function for these proteins is based on the collective results of all these programs (Fig. 1).

## Results and Discussion

### Physico-chemical parameters

Assigning the function of HPs bridges the gap in the knowledge of protein structure-function relationship and may reveal information about novel pathways responsible for pathogenesis. Based on the structural and functional information, they can be used as a drug targets or biomarkers for disease identification. Using different online servers, we tried to annotate the HPs present in genome of *F. nucleatum* subsp. *nucleatum*. Predicted physico-chemical parameters of all uncharacterized protein/HP are tabulated in Supplementary Table 3. These parameters provide an insight into the protein, such as pI value, extinction coefficient, etc. HPs with following accession IDs Q8RDL8, Q8REK2, Q8RH50, and Q8RIC0 have not shown extinction coefficient value due to absence of cysteine, tryptophan, and tyrosine residue. Most of the proteins are hydrophilic in nature as they have low GRAVY value. Forty-three percent of the total uncharacterized proteins had the acidic pH (pH < 7) while the rest 57% were basic (pH ≥ 7) in nature.

### Localization

**Fig. 2.**
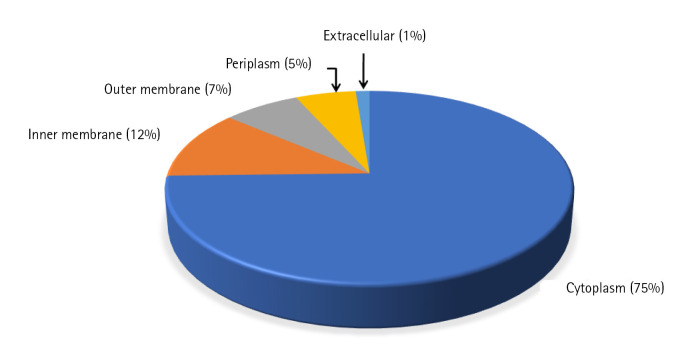
Sub-cellular localization. Pie-chart depicting the localization of uncharacterized proteins in *Fusobacterium nucleatum* as determined by the Cello server.

Cello server does not rely solely on the homology of the sequences but on the combination of two-level support vector machine classifiers to determine the subcellular location and thus, reduces the bias while increasing the accuracy [10]. Among 398 uncharacterized proteins, most of the proteins (74%) were predicted to be localized in cytoplasm whereas 12% and 7% of proteins were localized in the inner and outer membrane of the cell respectively. Only 1% of the proteins were found to be localized extracellular and 6% of proteins were localized in periplasm (Fig. 2, Supplementary Table 4). Presence of a signal peptide in a protein determines the translocation of protein inside or outside the cell. Signal peptide prediction was done using the SignalP 5.0 server which predicts its presence along with the location of their cleavage sites in bacterial proteins [27]. A total of 47 proteins were predicted to have signal peptide at their N-terminal, among which 30 proteins have standard signal peptide cleaved by signal peptidase I and the rest (17) have lipoprotein signal peptide cleaved by signal peptidase II (SPII) (Supplementary Table 4). One hundred forty-one proteins were predicted to be transmembrane proteins which might be involved in transport and signal transduction. Transmembrane proteins, especially the outer membrane proteins of the gram-negative bacteria behave as virulence factors and also help the pathogen in escaping defence mechanism of host [28].

### Domain identification

Annotation of uncharacterized proteins using InterProScan, Motif, SMART, HMMER, NCBI CDART, and BlastP search led to the identification of 90 proteins having functional domains (Supplementary Table 5). For increasing the accuracy of the results, we assigned the probable function to only those protein sequences whose conserved domains were predicted by two or more databases. As per this convention, out of 90 HPs with functional domains, functions were successfully assigned to 39 proteins with high confidence (Table 1) and other 7 proteins with relatively low confidence (Table 2).

### Predicted function

**Fig. 3.**
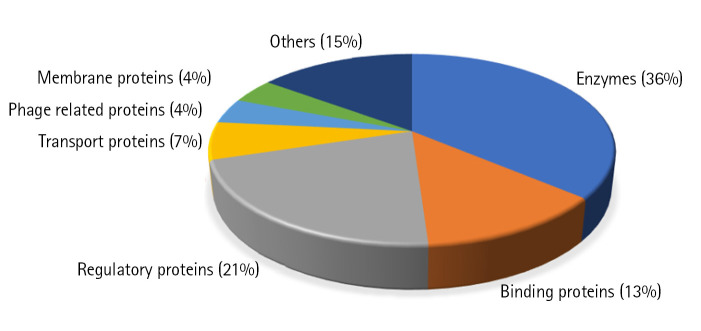
Probable functions of uncharacterized proteins. Chart shows the probable functions of uncharacterized proteins predicted based on domain and motif identification.

We could predict the function of 46 HPs out of which 17 proteins (37%) are enzymes, 5 (13%) are binding proteins, 10 regulatory proteins (21%), 3 transport proteins (6%), 2 are phage related (4%), 2 are membrane proteins (4%), and 7 proteins are involved in other functions (15%) (Fig. 3).

### Enzymes

Enzymes are the proteins that catalyze various metabolic pathways essential for the survival of an organism. *F. nucleatum* uncharacterized proteins with the following accession ID were identified as enzymes: Q8R669, Q8REG3, Q8REM4, Q8RFF3, Q8RFU1, Q8RGP8, Q8RH78, Q8RHS6, Q8RII7, Q8RE80, Q8RF13, Q8RFA9, Q8RHH4, Q8RI95, Q8RER1, Q8REJ6, and Q8REI4.

Q8R669 belongs to a nucleoside phosphorylase superfamily involved in S-adenosylmethionine mediated reaction. Enzymes of this family play a vital role in biofilm formation and pathogenesis of an organism. Q8R699 might be involved in these functions and can be used as a drug target for antimicrobial treatment [29].

Q8REG3 is D-component of 2-hydroxyglutaryl-CoA dehydratase (HGD-D) which undergoes dehydration to form enoyl CoA for the fermentation of α-amino acid. HGD consist of 2 components: component A which acts as an activator and component D which is a dehydratase enzyme. component A transfers electron to component D which in turn transfers electron to its substrate and thus perform the elimination of hydroxyl group [30].

Q8REM4 is a PGAP-1 like protein which encodes for glycosylphosphatidylinositol (GPI) inositol deacylase responsible for deacylation and transport of GPI-anchored protein from endoplasmic reticulum to Golgi [31].

Q8RFF3 and Q8RF13 are L,D-transpeptidase (LDT) enzymes which catalyze the peptide bond formation between two adjacent meso-diaminopimelic acid resulting in peptide cross-linking during synthesis of peptidoglycan cell wall. LDT can be used as a drug target as it has a role in cell wall synthesis which is essential for survivability of bacteria [32].

Q8RFU1 is a LpxI metal dependent hydrolase that catalyzes water mediated hydrolysis of β-phosphate of UDP-2,3-diacylglucosamine into LipidX in lipid A biosynthesis. Lipid A is essential for pathogenesis and viability of bacteria, thus, making Q8RFU1 a therapeutic target [33,34].

Q8RGP8 was identified as arginine deiminase, a homolog of DDAH (N,N-dimethylarginine dimethylaminohydrolases) which belongs to superfamily amidinotransferase. Arginine deiminase is involved in arginine metabolism in which NH_4_^+^ is produced. This NH_4_^+^ protects the bacteria from host acidic environment by raising the cytoplasmic pH. Based on the above function, this enzyme can be used as a probable drug target [35,36]. Q8RH78 is predicted to be an acyl-coenzyme A:6-aminopenicillanic acid acyl-transferase, known as the last enzyme that catalyzes the synthesis of antibiotic in penicillin biosynthesis pathway [37].

Q8RHS6 was characterized as a type IS restriction endonuclease that protects bacterial cell by recognizing and cleaving asymmetric sequences of bacteriophage DNA [38]. Q8RII7 was identified as a YmdB-like protein which belongs to calcineurin-like phosphatase/phosphodiesterase family. YmdB contains binuclear metal center which helps in biofilm formation and motility regulon expression [39].

Q8RE80 is an O-antigen ligase required for O-antigen ligation reaction in which lipid A attaches to core oligosaccharide and O antigen for the formation of lipopolysaccharide layer [40].

Q8RFA9 is a DNA repair enzyme protecting cell from the cytotoxic and mutagenic alkylating agents [41].

Q8RHH4 belongs to the PD-(D/E)XK nuclease superfamily 9 involved in a variety of functions such as DNA restriction, repair, modification, tRNA splicing, transposon excision, Holliday junction resolving, Pol I termination, etc. [42].

Q8RI95 is a permuted papain-like amidase enzyme thought to be involved in host-pathogen interactions and could be a potential drug target [43]. Q8RER1 is identified as small electron transfer protein known as flavodoxin. Flavodoxin proteins contain a non-covalently bonded flavin mononucleotide molecule as co-factor which also acts as a redox site [44]. This protein is involved in different metabolic pathways like nitrogen fixation and has the potential to be used as a therapeutic target [45].

Q8REJ6 was characterized as a thioredoxin protein having a conserved ‘thioredoxin motif’. Thioredoxins are involved in transferring of electrons from NADPH to thioredoxin via thioredoxin reductase. Thioredoxins have a role in DNA synthesis, protein repair, sulfur assimilation and in oxidative stress [46]. Q8REI4 is a cysteine protease PrP, responsible for cleaving L27 protein for efficient functioning of ribosome. Defective PrP leads to uncleaved L27 protein resulting in inhibition of bacterial growth [47].

### Regulatory proteins

A total of 10 proteins (Q8RDY5, Q8REC7, Q8RFD4, Q8RG23, Q8RHQ2, Q8RID9, Q8REK7, Q8RGG0, Q8RF29, and Q8REE9) were identified as regulatory proteins performing different functions.

Q8RDY5 is a translocon component of type I secretion system which enhances the serine sensitivity in a cell as serine is known to cause the inhibition of bacterial growth [48]. Q8REC7 was identified as a Cas7 or DevR protein which along with DevS has a regulatory role in fruiting body development in *Myxococcus xanthus* [49]. Q8RFD4 may act as a RelB regulatory protein which is an anti-toxin component of type I toxin-antitoxin complex. RelB inhibits RelE (toxin) functioning and binds to Rel operator thus allowing the transcriptional auto regulation [50]. Q8RG23 protein was identified as a ParD antitoxin protein, cognate of ParDE toxin-antitoxin system. ParE toxin inhibit the DNA synthesis and cell growth of bacteria. This activity of ParE is prevented by ParD antitoxin which performs dual function. N-terminal domain of ParD binds to DNA by Ribbon-Helix-Helix fold whereas C-terminal domain binds to its cognate part i.e., ParE antitoxin [51,52].

Q8RHQ2 protein belongs to a macro domain family, mainly present in pathogenic bacteria, archaea, single stranded viruses, and eukaryotes. Proteins with this domain, have diverse roles in regulation of ADP-ribosylation, DNA repair, and transcriptional regulation. Bacterial exotoxin mediates the ADP-ribosylation in target protein of host cell, thus contributing to the onset of infection [2].

Q8RID9 was predicted to be a RecG helicase which is a double-stranded DNA translocase. RecG regulates DNA transcription and avoids origin-independent pathological DNA synthesis by targeting Holliday junctions, three strand junction, R-loops, and D-loops [53,54]. This protein also possesses a Schlafen domain which binds to DNA and is involved in various functions such as DNA metabolism, DNA repair, and protecting cell from foreign elements [55]. Q8RF29 and Q8REK7 are the transcriptional repressor DNA binding winged-helix-turn-helix proteins belonging to Rrf2 transcriptional regulator family. Transcriptional repressor proteins contain [2Fe-2S]^+^ cluster which can repress the expression of the gene encoding for the Fe-S cluster assembly protein [56]. RseC/MucC is a transcriptional regulator localized in the inner membrane of the cell. Q8RGG0 is identified as RseC/MucC protein which positively regulates the sigma (RpoE) transcription factor. RpoE plays important role in regulating the gene expression of proteins having extracellular functions [57].

Q8REE9 is a FtsL/DivC protein involved in bacterial cell division. FtsL is a short protein which forms complex with 11 other proteins engaged in the synthesis of peptidoglycan wall [58].

### Binding proteins

We have identified five proteins (Q8RDP1, Q8RF86, Q8RGQ9, Q8RG53, and Q8RF83) involved in DNA, RNA, and protein binding. Q8RDP1 has a β-propeller domain that belongs to Kelch-repeat superfamily. Based on the function of Kelch-repeat, this protein is involved in a range of functions such as transcriptional and cytoskeletal regulation, signal trafficking and can also act as a substrate adapter for E3 ubiquitin ligase [59]. Q8RF86 was identified as a DNA helicase that catalyzes the separation of double stranded DNA when bound to a specific sequence in an ATP- dependent process [60]. Q8RGQ9 protein catalyzes ATP-dependent phosphorylation of 4-carbon acid sugars and nucleotides by binding them through N & C terminal domains respectively [61]. Q8RG53 and Q8RF83 contain a tetratricopeptide repeat and it has been identified that proteins with this repeat are involved in virulence related functions [6,62].

### Transport proteins

Q8RGC0, Q8RHR3, and Q8RH12 were predicted as transport proteins. Q8RGC0 is a PelG protein involved in transport of polysaccharides outside the cell for the synthesis of the biofilm. Formation of biofilm causes resistance to anti-microbial treatment and host defense mechanism thus increasing the survival chance of bacteria [63]. Q8RGC0 can also serve as a therapeutic target against *F. nucleatum* infection. Q8RHR3 contains a HEAT/Armadillo repeat which is present in nuclear protein transport complex [64]. Q8RH12 belongs to an ABC transporter family that transports nickel and cobalt. These transition metals act as cofactor for prokaryotic enzymes which are involved in various metabolic processes [65].

### Membrane proteins

Q8RE69 and Q8RGB9 are the only proteins that were identified as membrane proteins. Q8RE69 possesses a glycine zipper domain responsible for right-handed helix packing in the structure of membrane protein [66] whereas Q8RGB9 was predicted as a PagP β-barrel outer membrane protein of gram-negative bacteria. OMPLA and PagP are the two β-barrel protein enzymes involved in bacterial lipid metabolism. PagP-mediated lipid metabolism promotes infection by providing the resistance to antimicrobial peptides [67].

### Phage-related protein

Q8REC4 and Q8REB2 proteins were recognized as the phage resistance proteins. These proteins may form a part of abortive infection system which is involved in degradation of phage mRNA. This degradation of phage mRNA is brought about by halting the synthesis of phage proteins or by activation of the intracellular sensors that activate other proteins of further pathways for the abolishment of phage infection [68,69].

### Other proteins

Q8REQ3 protein of *F. nucleatum* belongs to SatD (secretion and acid tolerance) family having a role in acid resistance. This acid resistance may act as a virulence factor for survival of cariogenic bacteria [70,71]. Proteins Q8R6K0, Q8RGM7, and Q8RIP2 were predicted to be adhesion protein FadA, which is known to be involved in attachment and invasion of the host cells [72].

Q8RER4 was found to be involved in biosynthesis of colicin V, a bacteriocin, secreted by some bacteria for the intake of essential nutrients by inhibiting the growth of related strains [73]. Q8REC6 belongs to the CRISPR-Cas’s system involved in defense mechanism of prokaryotes against foreign substances [49]. Q8RHE9 protein was identified as a PilN, subunit of type IV pili which is well known for cell attachment, biofilm formation, twitching motility, and pathogenesis. PilN subunit upon binding with PilM causes a structural change in PilM and induces the type IV pilus system function [74].

### String analysis

String database contains the information on the functional and physical partners of the protein in a cell. Out of the 40 proteins searched on the string database, we got 30 with the confidence score > 1. Out of these 30 proteins, 9 had the confidence score ≥ 2.5 and had the maximum interacting partners.

Q8RDP1, Q8REC7, Q8RER4, Q8RFU1, Q8RGC0, Q8RHE9, Q8RHQ2, and Q8RE80 showed the maximum interacting partners with Q8RHQ2 having 29 interactions (Supplementary Table 6).

### Homology detection with the human proteins

Using protein BLAST to search for the homologous proteins in humans (*Homo sapiens* [taxid:9606]) revealed that out of the 36 annotated uncharacterized proteins only 1 (Q8RHQ2) has homology with any human protein. Rest 45 proteins have no similarity and can be used as probable drug target. Interestingly, on searching the DrugBank database for target sequences, out of all annotated proteins, only one protein which had similarity to *F. nucleatum* protein Q8RHQ2 showed interaction with several drugs. No other protein was listed in the DrugBank database.

### Structure prediction and modeling

Out of the 25 proteins (Table 3) for which we could find templates for homology modeling, we analyzed the structure of few important proteins. Three proteins Q8RGM7, Q8RHQ2, and Q8RII7 shared maximum homology with their respective templates (93%, 49%, and 50%, respectively). Another six functionally important proteins based on their annotation were also modeled.

**Fig. 4.**
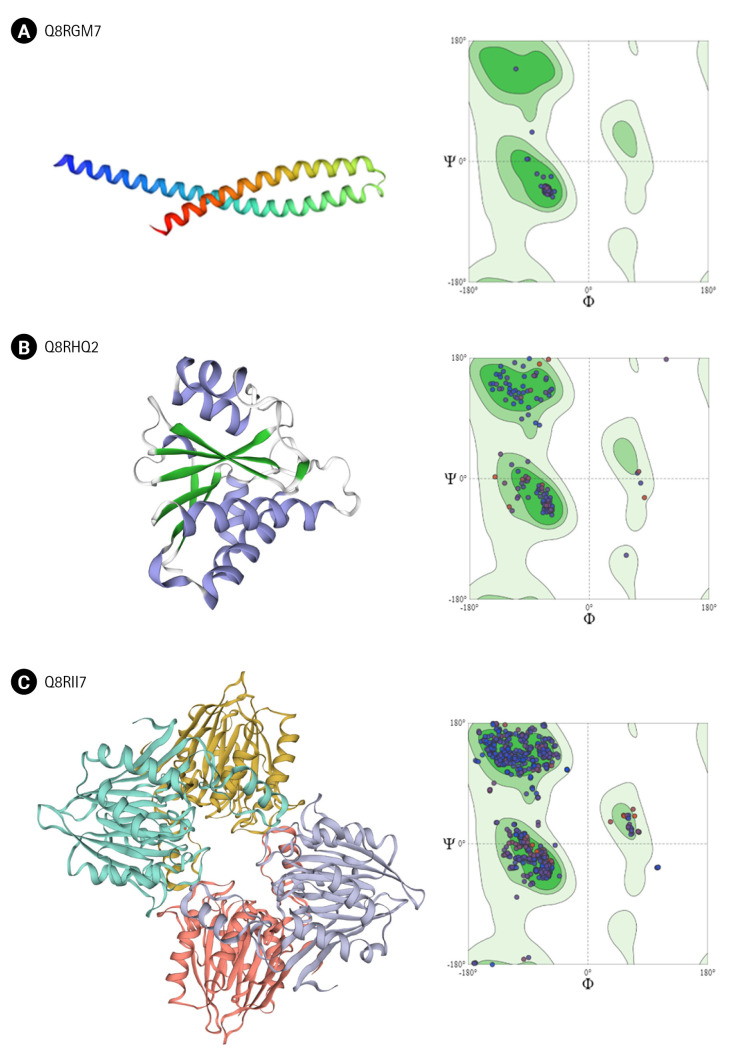
Structure modeling. Homology structure modeling and Ramachandran analysis of 3D structure using the Swiss-Model and PROCHECK were done for Q8RGM7 (A), Q8RHQ2 (B), and Q8RII7 (C).

Q8RGM7 shares about 93% identity with the template protein and the model was prepared with 99.75% confidence. The structure showed two large α-helices running antiparallel to each other joined by a loop. FadA exists in two forms in *F. nucleatum*; as 129 amino acid long non-secreted pre-FadA and 111 amino acid long mature secreted FadA. Ramachandran analysis shows all residues of the model in favorable region (Fig. 4A).

Q8RHQ2 was identified as macrodomain containing protein with a function in ADP ribosylation. The structural modeling with Phyre2 returned the template as 4IQY which records the structure of a human protein-proximal ADP-ribosyl-hydrolase MacroD2 [75]. Q8RHQ2 shares 49% sequence identity with the template protein. The final structure model comprised 172 amino acids containing 5 α helices and 6 β sheets. Ramachandran plot analysis showed 98.7% residues in allowed regions (Fig. 4B).

Q8RII7 is annotated as YmdB-like protein belonging to calcineurin-like phosphatase/phosphodiesterase family which helps in biofilm production. Phyre2 search identified crystal structure of *Bacillus subtilis* YmdB (4B2O) [39] as the template for the modeling with 50% identity to the query sequence. Similar to the template structure, the Q8RII7 model contained the conserved αββα architecture which is crucial for the co-ordination of two divalent metal ions. PROCHECK analysis showed 99.6 % residues in allowed regions and only 0.4% in disallowed region of Ramachandran plot (Fig. 4C).

Q8RFU1 was modeled based on template with PDB id 4GGM (LpxI from *Caulobacter crescentus*) [33]. The prediction model was seen as a dimer. Q8RGP8 structure was obtained using the structure of ArgZ (PDB id: 6JUY), an arginine dehydrolase enzyme found in the ornithine-ammonia cycle in cyanobacteria [76]. Q8RGP8 also takes the 5-fold-pseudosymmetric structural arrangement which is the signature motif of guandino-group modifying enzyme superfamily. Our annotation has identified Q8RGP8 as arginine deaminase enzyme. Q8RHS6 homology model was prepared using the template PDB 2EWF containing the structure of the larger subunit N.BspD6I of restriction endonuclease of *Bacillus* sp. [77]. Q8REI4 model was based on the PDB 2G0I showing the crystal structure of protein SMU.848 having unknown function from *Streptococcus mutans* [78]. Q8RFD4 has been annotated as a regulatory protein and its structure was predicted using a homologous structure of AvtR, a novel transcriptional regulator from a hyperthermophilic archaeal Lipothrixvirus (PDB 4HV0) [79]. Q8RG23 was modelled on the structure of ParE SO Cop-A SO toxin-antitoxin system (PDB 7ETR) of *Shewanella oneidensis* [80] (Supplementary Fig. 1A–1F).

### Virulence prediction

**Fig. 5.**
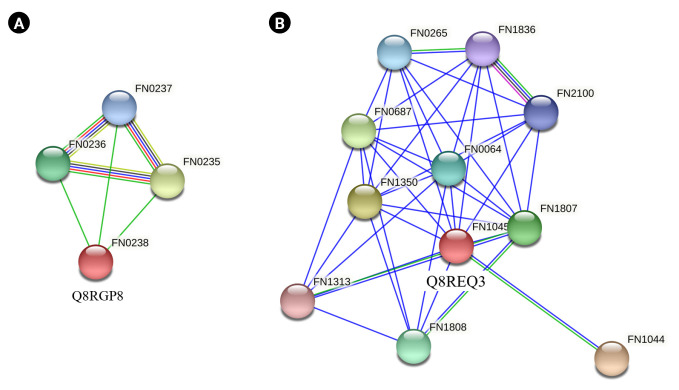
Interaction analysis. STRING database was searched to identify the interacting partners of the Q8RGP8 (A) and Q8REQ3 (B). The colored nodes indicate the query proteins and the first shell of interactors, the white nodes indicate the second shell of interactors, the empty nodes represent proteins with an unknown three-dimensional structure, and the filled nodes represent proteins with a known or predicted three-dimensional structure.

Q8RGP8 and Q8REQ3 were the common proteins listed as virulence factors by both VICMpred and VirulentPred software. Q8RGP8 is annotated as Arginine deiminase which is important for defence mechanism of the bacteria. Arginine deiminase increased expression has been found to be associated with the antibiotic resistance in *Streptococcus* bacteria by regulating the pH [81]. It has also been found to be important for survival of bacteria causing oral and dental infections [82,83]. String analysis of these two proteins revealed their interacting partners. Q8RGP8 interacts with three other neighboring proteins namely Q8RGQ3 (FN0235) which is an ABC transporter ATP binding protein, Q8RGQ0 (FN0236), an ATP transporter substrate binding protein and finally Q8RGP9 (FN0237), an ABC transporter permease protein (Fig. 5A). Arginine is known to be transported through a specialized ATP dependent transport system in *Escherichia coli* [84]. Similar system could also be present in the *F. nucleatum* which can be a lucrative drug target. Q8REQ3 is identified as SatD family protein having a role in acid resistance. Acid tolerance is essential for the survival of the cariogenic bacteria which forms a biofilm on teeth [71,85]. String analysis of Q8REQ3 displayed many interacting partners with this protein. Q8RE18 (FN1313) is one such protein which is an oligopeptide binding protein oppA which is involved in quorum sensing (Fig. 5B). Social behavior of the bacteria in biofilms is found to be regulated by quorum sensing proteins and thus the interaction of Q8REQ3 with the oppA protein is of much significance [86]. The homology model of Q8RGP8 has already been discussed in section above.

The functional annotation of non-characterized genes using recent software and programs provides new insight into the probable functions of previously uncharacterized proteins. *F. nucleatum* strain ATCC 25586 genome has 398 uncharacterized proteins and we annotated 46 out of them. Physico-chemical parameter determination was done for all the uncharacterized proteins leading to prediction of their pI and approximate molecular weight. About 43% of the proteins were shown to be acidic with pH of less than 7 while 53% have pH of more than 7. Subcellular location of the proteins is an important determinant of its function, and it was predicted using the Cello, SignalP 5.0, and TMHMM servers. The majority of the proteins (75%) were seen to be localized in cytoplasm while 12% were outer membrane proteins. Searching for conserved sequential and structural features (domains and motifs) using the combined results obtained from InterProScan, Motif, SMART, HMMER, and NCBI-CDART programs identified 17 proteins as enzymes, 10 have regulatory roles, 5 as binding proteins and final 7 have other functions as transport, membrane proteins, etc. Another seven proteins were also annotated based on the result of at least three of these programs. ROC analysis of the software and programs used for annotation show a reliable confidence on the approach. Structural modeling was performed for 9 proteins for which suitable templates with good homology were obtained using the Swiss-Model and Phyre2 servers. Two probable virulence-related proteins (Q8RGP8 and Q8REQ3) were also identified which provide excellent opportunity for their further detailed analysis as potent drug targets. This study has resulted in the identification of many interesting proteins which were previously mentioned as uncharacterized and can provide deep understanding of the biology of the pathogen with related experimental study.

## Figures and Tables

**Table 1.** List of *Fusobacterium nucleatum* uncharacterized proteins annotated with high confidence

S. No.	Accession ID	Identified protein
1	Q8RDP1	Kelch-repeat superfamily Protein
2	Q8RDY5	Translocon component of type Ⅲ secretion system
3	Q8RE69	Glycine zipper domain containing protein
4	Q8REC7	Cas7 or DevR protein
5	Q8REG3	2-Hydroxyglutaryl-CoA dehydratase (HGD-D)
6	Q8REM4	PGAP-1 like protein
7	Q8RER4	Colicin V protein
8	Q8RFD4	RelB regulatory protein
9	Q8RFF3	L,D-transpeptidase
10	Q8RFU1	LpxI metal dependent hydrolase
11	Q8RGC0	PelG protein
12	Q8RGM7	Adhesion protein FadA
13	Q8RGP8	Arginine deiminase
14	Q8RGQ9	Four-carbon acid sugar kinase family protein
15	Q8RH78	Acyl-coenzyme A:6-aminopenicillanic acid acyl-transferase (AAT)
16	Q8RHE9	PilN protein
17	Q8RHQ2	Macro domain family protein
18	Q8RHR3	HEAT/Armadillo repeat protein
19	Q8RHS6	Type IS Restriction endonuclease
20	Q8RII7	YmdB like protein
21	Q8RE80	O-antigen ligase protein
22	Q8REK7	DNA binding winged-helix-turn-helix protein
23	Q8REQ3	SatD family protein
24	Q8RIP2	Adhesion protein FadA
25	Q8RF13	L,D-transpeptidase
26	Q8REC4	Phage resistance protein
27	Q8RFA9	DNA repair enzyme
28	Q8RHH4	PD-(D/E) XK nuclease
29	Q8REE9	FtsL/DivC protein
30	Q8REB2	Phage resistance protein
31	Q8RER1	Flavodoxin protein
32	Q8REJ6	Thioredoxin
33	Q8RGB9	PagP β-barrel protein
34	Q8RF83	Tetratricopeptide (TPR) repeat protein
35	Q8REI4	Cysteine protease PrP
36	Q8REC6	CRISPR-Cas protein
37	Q8RGG0	RseC/MucC protein
38	Q8RF29	DNA binding winged-helix-turn-helix protein
39	Q8R6K0	Adhesion protein FadA

**Table 2.** List of *Fusobacterium nucleatum* uncharacterized proteins with low confidence

S. No.	Accession ID	Identified protein
1	Q8R669	Nucleoside phosphorylase
2	Q8RF86	DNA helicase
3	Q8RG23	ParD antitoxin protein
4	Q8RID9	RecG helicase
5	Q8RG53	Tetratricopeptide (TPR) repeat protein
6	Q8RI95	Permuted papain-like amidase
7	Q8RH12	ABC transporter family protein

**Table 3.** List of protein structures from Protein Data Bank used as templates for homology modelling of the uncharacterized proteins of *Fusobacterium nucleatum*

S. No.	Accession Id	Template	Sequence identity
1	Q8RDP1	5YY8	18.9
2	Q8RE69	7MUS	33.9
3	Q8REC7	7TR9	17.8
4	Q8REM4	6WPY	15.5
5	Q8RF86	3LMM	14.1
6	Q8RFD4	4HV0	19.1
7	Q8RFU1	4GGM	26.1
8	Q8RG23	7ETR	26.8
9	Q8RGM7	3ETW	97.3
10	Q8RGP8	6JUY	17.2
11	Q8RGQ9	4XFM	22.4
12	Q8RH78	2X1C	17.1
13	Q8RHQ2	4IQY	49.7
14	Q8RHR3	4RV1	16.2
15	Q8RHS6	2EWF	19.2
16	Q8RID9	3LMM	19.3
17	Q8RII7	4B20	50.4
18	Q8REK7	7BOC	19.1
19	Q8RIP2	3ETW	27.9
20	Q8RER1	5MJI	19.5
21	Q8REI4	2G0I	20.9
22	Q8RH12	4HZU	22.8
23	Q8RGG0	6RAJ	20.3
24	Q8RF29	7B0C	22.4
25	Q8R6K0	3ETW	19.6

## Supplementary Materials

Supplementary Table 1. List of 50 proteins with known function from *Fusobacterium nucleatum* used for receiver operating characteristics analysis

Supplementary Table 2. Receiver operating characteristics (ROC) result of various tools used for function prediction

Supplementary Table 3. Predicted physicochemical properties of the uncharacterized proteins from *Fusobacterium nucleatum* using ProtParam

Supplementary Table 4. Predicted sub-cellular localization (Cello server), signal peptide (SignalP 5.0) and transmembrane helices (TMHMM) for the list of *Fusobacterium nucleatum* uncharacterized proteins of *F. nucleatum*

Supplementary Table 5. Functional domains identified in uncharacterized proteins of *Fusobacterium nucleatum*

Supplementary Table 6. Protein-protein interaction of uncharacterized proteins from *Fusobacterium nucleatum* ATCC 25586

Supplementary Fig. 1. Structural modeling. Homology based structure prediction and Ramachandran analysis of Q8RFU1 (A), Q8RGP8 (B), Q8RHS6 (C), Q8REI4 (D), Q8RFD4 (E), and Q8RG23 (F).
