# Supplementary material for: Functional annotation of uncharacterized proteins from Fusobacterium nucleatum: identification of virulence factors
Source: Genomics Inform. 2023 Jun 30;21(2):e21. doi: 10.5808/gi.22065 (PMC10326533; doi:10.5808/gi.22065)
Supplement: Supplementary Table 1. — List of 50 proteins with known function from Fusobacterium nucleatum used for receiver operating characteristics analysis [file gi-22065-Supplementary-Table-1.pdf]

Supplementary Table 1. List of 50 proteins with known function from *Fusobacterium nucleatum* used for receiver operating characteristics analysis

S. No.	Accession ID	Protein name known function	InterProScan		MOTIF		SMART		HMMER		CDART	
			Prediction	Score	Prediction	Score	Prediction	Score	Prediction	Score	Prediction	Score
1	Q8RIH0	30S Ribosomal Protein	Ribosomal S8	1 (4)	Ribosomal Protein S8, YjcQ Protein, LacI family	1 (4)	Ribosomal_S8	1 (4)	Ribosomal Protein S8	1 (4)	Ribosomal Protein S8	1 (4)
2	Q8RG98	ATP-dependent 6-Phosphofructokinase	ATP-dependent 6-Phosphofructokinase, prokaryotic type	1 (5)	Phosphofructokinase, ATP-NAD kinase N-terminal domain, Diacylglycerol kinase catalytic domain	1 (4)	Phosphofructokinase	1 (4)	Phosphofructokinase	1 (4)	Phosphofructokinase	1 (4)
3	P58819	Putative zinc metalloprotease	Peptidase M50, Putative membrane-associated zinc metallopeptidase, PDZ domain	1 (4)	Peptidase family M50, PDZ domain, Peptidase M50B-like, Putative zincin peptidase	1 (4)	PDZ Domain	1 (3)	Peptidase family M50, PDZ domain	1 (4)	Membrane-associated protease RseP	1 (3)
4	Q8RFX6	Probable D-serine dehydratase	D-serine ammonia-lyase , Pyridoxal-phosphate dependent enzyme, Tryptophan synthase beta subunit-like PLP-dependent enzyme	1 (4)	Pyridoxal-phosphate dependent enzyme	1 (3)	PALP	1 (3)	Pyridoxal-phosphate dependent enzyme (PALP)	1 (3)	Tryptophan synthase beta II superfamily	1 (3)
5	Q8RGD8	ATP synthase subunit	ATP synthase, F0 complex, subunit b, bacterial	1 (5)	ATP Synthase B/B’ CF (0), Mitochondrial ATP synthase B chain precursor	1 (5)	Mt_ATP-synt_B	1 (4)	ATP synthase B/B’	1 (5)	ATP synthase subunit B	1 (5)
6	Q8RGM1	GTPase Era	GTP binding domain, GTP-binding protein Era, P-loop containing nucleoside triphosphate hydrolase, Era-type	1 (5)	50S Ribosome-binding GTPase, KH domain	1 (4)	FeoB_N, KH_2	1 (3)	50S ribosome binding GTPase, KH domain	1 (4)	GTPase Era	1 (5)

			guanine nucleotide-binding (G) domain, K homology domain-like, alpha/beta, K homology domain, type 2, Small GTP-binding protein domain,									
7	Q8RDM3	Methionyl-tRNA formyltransferase	Methionyl-tRNA formyltransferase	1 (5)	Formyl transferase, Formyl transferase C	1 (4)	Formyl transferase N, Formyl transferase C	1 (4)	Formyl transferase, Formyl transferase C	1 (4)	Methionyl-tRNA formyltransferase	1 (5)
8	Q8RIA6	Undecaprenyl-diphosphatase	Undecaprenyl-diphosphatase UppP	1 (5)	Bacitracin resistance protein BacA, Lipoma HMGIC fusion partner-like protein	1 (3)	Bacitracin resistance protein BacA	1 (3)	Bacitracin resistance protein BacA	1 (3)	Bacitracin resistance protein BacA	1 (3)
9	Q8RF40	ABC transporter ATP-binding Protein	ABC transporter-like ATP binding domain, AAA+ ATPase domain	1 (5)	ABC transporter, AAA domain, Putative AbiEii toxin, Type IV TA system	1 (4)	AAA	1 (3)	ABC Transporter	1 (4)	ABC-type uncharacterized transport system	1 (3)
10	Q8RDX5	COMF operon protein 3	Phosphoribosyl transferase domain, Phosphoribosyltransferase-like	1 (2)	Phosphoribosyl transferase domain, Uracil phosphoribosyltransferase, UTP 11 protein	1 (2)	Phosphoribosyl transferase domain	1 (2)	Phosphoribosyl transferase domain	1 (2)	Phosphoribosyl transferase Type I domain	1 (2)
11	Q8RGV1	Cell division inhibitor MinD	ATP binding protein MinD, ATP binding protein MinD/FleN, AAA domain, P-loop containing nucleoside triphosphatase	1 (3)	AAA domain, CobQ/CobB/MinD/ParA nucleotide binding domain, NUBPL iron-transfer P-loop NTPase, ATPase MipZ	1 (3)	ArsA_ATPase	1 (2)	AAA Domain	1(3)	Septum formation inhibitor-activating ATPase MinD	1 (5)

12	Q8RGB3	Polysaccharide Deacetylase	NodB homology domain, Glycoside hydrolase/deacetylase, beta/alpha-barrel, Glycosyltransferase subfamily 4-like, N-terminal domain	1 (2)	Polysaccharide deacetylase, Glycosyltransferase Family 4	1 (5)	Glycosyltransferase_4, Polysaccharide Deacetylase_1	1 (5)	Glycosyltransferase family 4, Polysaccharide Deacetylase	1 (5)	Glycosyltransferase GTB type superfamily, CE4_SF superfamily	1 (2)
13	Q8R676	Methyltransferase	S-adenosyl-L-methionine-dependent methyltransferase, Methyltransferase domain	1 (5)	Methyltransferase domain	1 (5)	Methyltransf_32	1 (5)	Methyltransferase domain	1 (5)	Adenosylmethionine-dependent methyltransferases	1 (4)
14	Q8RHG6	Amino acid carrier protein alsT	Sodium:alanine symporter	1 (4)	Sodium:alanine symporter family	1 (4)	Sodium:alanine symporter family	1 (4)	Sodium:alanine symporter family	1 (4)	Sodium:alanine symporter family	1 (4)

15	Q8R6C5	Cysteine desulfurase	Pyridoxal phosphate-dependent transferase, small domain, Aminotransferase class-V, pyridoxal-phosphate binding site, Pyridoxal phosphate-dependent transferase, Cysteine desulfurase NifS, bacterial/archaeal, Pyridoxal phosphate-dependent transferase, major domain, Aminotransferase class V domain, Cysteine desulfurase	1 (5)	Aminotransferase class-V, Beta-eliminating lyase, Cys/Met metabolism PLP-dependent enzyme, Pyridoxal-dependent decarboxylase conserved domain, DegT/DnrJ/EryC1/StrS aminotransferase family, Aminotransferase class I and II	1 (4)	Aminotran_1_2	1 (2)	Aminotransferase class V	1 (4)	Aspartate aminotransferase (AAT) superfamily	1(3)

16	Q8REI0	Long-chain-fatty-acid—CoA ligase	ACP-like superfamily, AMP-binding, conserved site, Phospholipid/glycerol acyltransferase, ANL, N-terminal domain, AMP-binding, AMP-dependent synthetase/ligase, AMP-binding enzyme, C-terminal domain superfamily	1 (2)	AMP-binding enzyme	1 (2)	AMP-binding enzyme, Phosphate acyltransferase	1 (2)	AMP-binding enzyme, Acyltransferase	1 (2)	Adenylate forming domain, Class I superfamily, Phosphopantetheine binding superfamily, Lysophospholipid acyltransferases superfamily	1 (2)
17	Q8REA2	Cell division protein FtsI	Penicillin-binding protein, transpeptidase, Beta-lactamase/transpeptidase-like, Penicillin-binding protein, dimerisation domain superfamily, Penicillin-binding protein, dimerisation domain, Penicillin-binding protein 2	1 (3)	Penicillin binding protein transpeptidase domain	1 (3)	Penicillin binding protein_dimeristaion, Transpeptidase	1 (3)	Penicillin-binding protein dimerization domain, Penicillin-binding protein transpeptidase domain	1 (3)	Penicillin-binding protein 2	1 (3)
18	Q8REN2	Tetratricopeptide repeat family protein	Tetratricopeptide repeat, Tetratricopeptide-like helical domain superfamily	1 (5)	Tetratricopeptide repeat	1 (5)	TPR_11	1 (5)	TPR Repeat	1 (5)	PLN03088 Superfamily, Adventurous gliding motility	1 (3)

19	Q8RGC7	Iron (III)-transport system permease protein sfuB	ABC transporter type 1, transmembrane domain MetI-like, MetI-like superfamily	1 (2)	Binding protein dependent transport system inner membrane component	1 (2)	Binding protein dependent transport system inner membrane component	1 (2)	Binding protein dependent transport system inner membrane component	1 (2)	TPR repeat lipoprotein GltE ABC-type Fe3+ transport system, permease component	1 (4)
20	Q8REA5	Hemolysin	RNA-binding S4 domain superfamily, Haemolysin A /rRNA methyltransferase TlyA, RNA-binding S4 domain, Ribosomal RNA methyltransferase FtsJ domain, S-adenosyl-L-methionine-dependent methyltransferase	1 (4)	FtsJ-like methyltransferase, S4 domain, Ribosomal protein L11 methyltarnsferase , Methyltransferase domain	1 (3)	S4 RNA-binding domain	1 (3)	FtsJ-like methyltransfe rase	1 (3)	Predicted rRNA methylase YqxC	1 (3)

21	Q8RFB8	CBS domain containing protein	Winged helix-like DNA-binding domain superfamily, Winged helix DNA-binding domain superfamily, CBS domain, Protein of unknown function UCP026546, HtH-CBS, Helix-turn-helix, type 11	1 (5)	CBS domain, HTH domain, DeoR-like helix-turn-helix domain	1 (5)	HTH_11, CBS domain	1 (5)	HTH_11, CBS domain	1 (5)	HTH superfamily, CBS pair superfamily	1 (4)
22	Q8RIQ6	Hypothetical exported 24-amino acid repeat protein	MORN variant	1 (2)	MORN repeat variant, FAM194 protein	1 (2)	MORN_2	1 (2)	MORN repeat variant	1 (2)	YwqK superfamily	1 (2)
23	Q8R5X2	Multifunctional fusion protein	Peptide methionine sulphoxide reductase MsrA domain, Peptide methionine sulphoxide reductase MsrB domain, Thioredoxin-like superfamily, Mss4-like superfamily, Redoxin, Peptide methionine sulfoxide reductase MsrB, Thioredoxin domain	1(2)	Peptide methionine sulfoxide reductase, SelR domain, Redoxin	1 (2)	Redoxin, Peptide methionine sulfoxide reductase (PMSR), SelR domain	1 (2)	Redoxin, Peptide methionine sulfoxide reductase, SelR domain	1 (2)	PRK14018 superfamily	1 (2)

24	Q8REA6	Ribosome biogenesis GTPase A	P-loop containing nucleoside triphosphate hydrolase, GTP binding domain, GTP-binding protein, ribosome biogenesis, Circularly permuted (CP)-type guanine nucleotide-binding (G) domain, GTP-binding protein, orthogonal bundle domain superfamily, GTPase, MTG1	1 (4)	50S ribosome-binding GTPase	1 (4)	MMR_HSR1	1 (4)	50S ribosome-binding GTPase	1 (4)	GTPase_YlqF superfamily	1 (4)
25	Q8RE48	Zinc metallohydrolase, glyoxalase II family	Metallo-beta-lactamase, Ribonuclease Z/Hydroxyacylglutathione hydrolase-like	1 (3)	Metallo-beta-lactamase superfamily, ODP family beta lactamase	1 (3)	Lactamase_B	1 (3)	Metallo-beta-lactamase superfamily	1 (3)	Metallo-hydrolase-like_MBL-fold	1 (3)
26	Q8RFZ4	Hypothetical membrane-spanning protein	AbrB family, AbrB duplication	0 (2)	Transition state regulatory protein AbrB	0 (2)	AbrB	0 (2)	Transition state regulatory protein AbrB	0 (2)	AbrB superfamily	0 (2)
27	Q8R696	Serine racemase	PLP-binding barrel, D-serine dehydratase-like domain, Alanine racemase, N-terminal, D-serine dehydratase-like domain superfamily	1 (3)	Ala_racemase,N-terminal domain, Putative serine dehydratase domain	1 (3)	Orn_Arg_deC_N, D-ser dehydratase	1 (3)	Ala_racemase,N-terminal domain, Putative serine dehydratese domain	1 (3)	Dsd1 superfamily	1 (3)

28	Q8REC9	ATP-dependent RNA helicase	CRISPR-associated Cas3-type HD domain, Helicase Cas3, CRISPR-associated, core, P-loop containing nucleoside triphosphate hydrolase, CRISPR-associated endonuclease/helicase Cas3, Helicase superfamily 1/2, ATP-binding domain	1 (3)	HD domain, DEAD/DEAH box helicase	1 (3)	DEXDc, Helicase_C	1 (3)	HD domain	1 (2)	Cas3 superfamily	1 (3)
29	Q8RGZ3	Hemolysin	Filamentous haemagglutinin repeat, Hemagglutinin repeat, Pectin lyase fold/virulence factor	1 (4)	Hemagglutinin repeat	1 (4)	Filamentous Haemagglutinin,Parallel beta-helix repeat, SH3 domain	1 (4)	Filamentous Haemagluttin in repeat	1 (4)	FhaB superfamily	1 (2)
30	Q8RI30	Integral membrane protein	EamA domain	1 (3)	EamA-like transporter family	1 (3)	EamA	1 (3)	EamA-like transporter family	1 (3)	RhaT Superfamily	1 (3)
31	Q8REG6	Phosphonates transport system permease protein phnE	ABC transporter type 1, transmembrane domain MetI-like, MetI-like superfamily, Phosphonate ABC transporter, substrate-binding protein	1(4)	Binding-protein-dependent transport system inner membrane component	1 (3)	BPD_transp_1	1 (4)	Binding-protein-dependent transport system inner membrane component	1 (4)	Phosphonate ABC transporter, Permease protein PhnE	1 (5)

32	Q8R6A6	Single-stranded-DNA-specific exonuclease RecJ	DHH phosphoesterase superfamily, DHHA1 domain, DDH domain, Bacterial RecJ exonuclease, RecJ, OB domain	1 (4)	DHH family, RecJ OB domain, DHHA1 domain	1 (4)	DHH, DHHA1	1 (3)	DHH family, DHHA1 domain, RecJ OB domain	1 (4)	single-stranded-DNA-specific exonuclease RecJ	1 (5)
33	Q8RHU9	BAX Protein	Mannosyl-glycoprotein endo-beta-N-acetylglucosamidase-like domain	1 (2)	Mannosyl-glycoprotein endo-beta-N-acetylglucosaminidase	1 (2)	Glucosaminidase	1 (2)	Mannosyl-glycoprotein endo-beta-N-acetylglucosaminidase	1 (2)	Bax superfamily	1 (4)
34	Q8RGN1	Transcriptional repressor smtB	Winged helix DNA-binding domain superfamily, HTH ArsR-type DNA-binding domain, ArsR-type transcription regulator, HTH motif, Winged helix-like DNA-binding domain superfamily, ArsR-like helix-turn-helix domain	1 (2)	Bacterial regulatory protein, arsR family, Helix-turn-helix domain, Winged helix DNA-binding domain, MarR FAMILY	1 (2)	HTH_ARSR	1 (2)	Bacterial regulatory protein, arsR family	1 (2)	HTH_metalloregulator ArsR/SmtB family transcription factor	1 (3)
35	Q8RFN3	ABC transporter permease protein	MetI-like superfamily, ABC transporter type 1, transmembrane domain MetI-like	1 (3)	Binding-protein-dependent transport system inner membrane component	1 (3)	BPD_transp_1	1 (3)	Binding-protein-dependent transport system inner membrane component	1 (3)	Transmembrane subunit found in Periplasmic Binding protein (PBP)-dependent ATP-	1 (3)

											binding Cassette (ABC) transporters	
36	Q8RI53	DUF4261 domain-containing protein	Domain of unknown function DUF4261	1 (4)	Domain of unknown function (DUF4261)	1 (4)	DUF4261	1 (4)	Domain of unknown function DUF4261	1 (4)	DUF4261 superfamily	1 (4)
37	Q8RE20	Biopolymer transport exbD protein	Biopolymer transport protein ExbD/TolR	1 (4)	Biopolymer transport protein ExbD/TolR, Staphopain proregion, Family of unknown function (DUF5633), Surface adhesin CshA repetitive domain	1 (4)	ExbD	1 (4)	Biopolymer transport protein ExbD/TolR	1 (4)	ExbD superfamily	1 (4)
38	Q8R5Y6	MGTC/SAPB Family Membrane protein	MgtC/SapB/SrpB/YhiD family	1 (4)	MgtC family, Binding domain of Nse4/EID3 to Nse3-MAGE	1 (4)	MgtC	1 (4)	MgtC family	1 (4)	SapB superfamily	1 (4)
39	Q8REZ9	Phosphohydrolase (MUTT/NUDIX family Protein)	Coenzyme A pyrophosphatase, NUDIX hydrolase domain, NUDIX hydrolase-like domain superfamily	1 (4)	NUDIX domain, Family of unknown function (DUF6650)	1 (4)	NUDIX	1 (4)	NUDIX domain	1 (4)	Nudix_Hydroalse superfamily	1 (4)
40	Q8RHD3	Tricarboxylate transport membrane protein TctB	Protein of unknown function DUF1468	1 (3)	Tripartite tricarboxylate transporter TctB family, Phosphatidylinositol N-acetylglucosaminyltransferase	1 (4)	TctB	1 (4)	Tripartitie tricarboxylate transporter TctB family	1 (4)	TctB Superfamily	1 (4)

41	Q8RF01	T (6)A37 threonylcarbamoyladenosine biosynthesis protein TsaE	P-loop containing nucleoside triphosphate hydrolase, tRNA threonylcarbamoyl adenosine modification protein TsaE	1 (4)	Threonylcarbamoyl adenosine biosynthesis protein TsaE, AAA domain	1 (4)	TsaE	1 (4)	Threonylcarbamoyl adenosine biosynthesis protein TsaE	1 (4)	YlqF related GTPase superfamily	1 (2)
42	Q8REH7	LemA protein	LemA-like domain superfamily, MamQ/LemA	1 (4)	LemA Family, Ribosomal protein L36e	1 (4)	LemA	1 (4)	LemA	1 (4)	LemA Superfamily	1 (4)
43	Q8RI18	Hemolysin activator protein	Two partner secretion pathway transporter, Polypeptide-transport-associated, ShlB-type, Haemolysin activator HlyB, C-terminal, ShlB, POTRA domain	1 (4)	Haemolysin secretion/activation protein ShlB/FhaC/HecB	1 (3)	POTRA_2, POTRA_3, ShIB	1 (3)	POTRA domain,ShIB-type, Haemolysin secretion/activation protein ShIB/FhaC/HecB	1 (3)	FhaC Superfamily	1 (3)
44	Q8R628	Cobalt chelatase	Anaerobic cobalt chelatase	1 (4)	Cobalt chelatase (CbiK), CbiX	1 (5)	CbiX	1 (4)	Cobalt Chelatase, CbiK	1 (5)	CbiK superfamily	1 (4)
45	Q8R5P1	DNAse I homologous protein DHP2	Deoxyribonuclease I, Endonuclease/exonuclease/phosphatase superfamily	1 (4)	Endonuclease/Exonuclease/phosphatase family	1 (2)	DNaseIc	1 (3)	No result	0 (2)	EEP superfamily	1 (2)
46	Q8RFG2	Formiminotetrahydrofolate cyclodeaminase	Formimidoyltransferase-cyclodeaminase-like superfamily, Cyclodeaminase/cyclohydrolase	1 (4)	Formiminotransferase-cyclodeaminase, SMODS and SLOG-associating 2TM effector domain family 5	1 (4)	FTCD_C	1 (4)	Formiminotransferase-cyclodeaminase (FTCD_C)	1 (4)	FTCD_C superfamily	1 (4)
47	Q8RE19	Biopolymer transport exbB protein	MotA/TolQ/ExbB proton channel	1 (4)	MotA/TolQ/ExbB proton channel family, Domain of unknown function (DUF5671)	1 (4)	MotA_ExbB	1 (4)	MotA/TolQ/ExbB proton channel family	1 (4)	MotA_ExbB superfamily	1 (4)

48	Q8REQ8	Branched-chain amino acid transport protein azlC	Branched-chain amino acid transport, permease	1 (4)	AzlC protein, Protein of unknown function (DUF3242)	1 (4)	AzlC protein	1 (4)	AzlC protein	1 (4)	AzlC superfamily	1 (4)
49	Q8RGF7	Transport protein	Na+/H+ antiporter, NhaC-like, C-terminal, Putative Na+/H+ antiporter, N-terminal	1 (3)	Na+/H+ antiporter family, Citrate transporter, GntP family permease, Tripartite ATP-independent periplasmic transporter, DctM component, Inner membrane protein CreD	1 (3)	GntP_permease	1 (3)	Na+/H+ antiporter family	1 (3)	YuiF superfmaily	1 (3)
50	Q8R6J4	Transposase	Transposase, IS116/IS110/IS902, Transposase, IS111A/IS1328/IS1533, N-terminal	1 (4)	Transposase, Transposase IS116/IS110/IS902 family	1 (5)	DEDD_Tnp_IS110, Transposase_20	1 (4)	Transposase, Transposase IS116/IS110/IS902 family	1 (5)	IS110 family transposase	1 (4)
