# Supplementary material for: Functional annotation of uncharacterized proteins from Fusobacterium nucleatum: identification of virulence factors
Source: Genomics Inform. 2023 Jun 30;21(2):e21. doi: 10.5808/gi.22065 (PMC10326533; doi:10.5808/gi.22065)
Supplement: Supplementary Table 2. — Receiver operating characteristics (ROC) result of various tools used for function prediction [file gi-22065-Supplementary-Table-2.pdf]

Supplementary Table 2. Receiver operating characteristics (ROC) result of various tools used for function prediction

S. No.	Software	Accuracy (%)	Sensitivity (%)	Specificity (%)	ROC area
1	InterProScan	84	83.7	100	0.918
2	Motif	84	83.7	100	0.918
3	SMART	82	81.6	100	0.908
4	HMMER	84	83.3	100	0.917
5	NCBI CDART	84	83.7	100	0.918
6	Average	83.6	83.2	100	0.90
