# Supplementary material for: Functional annotation of uncharacterized proteins from Fusobacterium nucleatum: identification of virulence factors
Source: Genomics Inform. 2023 Jun 30;21(2):e21. doi: 10.5808/gi.22065 (PMC10326533; doi:10.5808/gi.22065)
Supplement: Supplementary Table 3. — Predicted physicochemical properties of the uncharacterized proteins from Fusobacterium nucleatum using ProtParam [file gi-22065-Supplementary-Table-3.pdf]

Supplementary Table 3. Predicted physicochemical properties of the uncharacterized proteins from *Fusobacterium nucleatum* using ProtParam

S. No.	Accession Id	Amino acids	Molecular weight (Da)	Theoretical PI	Extinction coefficient (/M/cm)	Instability index		Aliphatic index	Grand average of hydropathicity (GRAVY)
						Computed	Classification		
1	Q8R6J3	179	19,927.50	4.70	7,450	55.74	Unstable	73.24	–0.640
2	Q8R669	623	73,660.97	8.34	755,570	32.94	Stable	98.23	–0.328
3	Q8RDL8	29	3,251.10	9.70	-	22.94	Stable	178.28	1.579
4	Q8RDM0	192	22,257.01	9.02	13,410	45.61	Unstable	105.99	–0.381
5	Q8RDN3	111	12,542.45	5.71	2,980	37.83	Stable	48.65	–0.911
6	Q8RDP1	372	41,575.50	8.95	64,750	32.49	Stable	88.06	–0.351
7	Q8RDR8	45	5,373.33	4.96	5,960	34.70	Stable	114.89	0.313
8	Q8RDV4	56	6,853.47	7.72	6,085	41.21	Unstable	170.54	1.471
9	Q8RDW3	1176	131,023.66	9.28	104,980	26.52	Stable	78.48	–0.640
10	Q8RDY5	233	27,447.25	4.62	37,025	38.70	Stable	93.73	–0.487
11	Q8RE02	122	14,974.35	7.75	8,940	16.62	Stable	68.61	–0.763
12	Q8RE16	177	20,140.71	4.25	10,430	39.85	Stable	98.59	–0.314
13	Q8RE33	169	1,9231.66	5.61	27,975	23.79	Stable	79.47	–0.424
14	Q8RE35	393	46,940.93	7.06	81,600	29.17	Stable	75.93	–0.650
15	Q8RE36	453	52,702.40	5.35	69,110	27.17	Stable	83.27	–0.550
16	Q8RE37	411	48,040.53	6.92	73,120	26.76	Stable	86.03	–0.474
17	Q8RE38	340	39,706.35	8.18	65,670	25.45	Stable	92.29	–0.426
18	Q8RE39	571	67,587.95	5.67	88,145	30.06	Stable	88.70	–0.585
19	Q8RE61	120	13,773.53	4.62	20,065	28.23	Stable	94.17	–0.443
20	Q8RE69	58	5,249.99	7.96	0	16.37	Stable	96.21	0.543
21	Q8RE90	63	7,315.26	5.13	16,960	9.44	Stable	82.06	–0.438
22	Q8RE93	288	34,331.96	4.63	57,675	36.97	Stable	73.47	–0.621
23	Q8REA1	140	16,563.03	9.35	20,400	49.14	Unstable	139.93	1.009
24	Q8REA8	124	14,596.52	9.33	11,000	42.58	Unstable	124.92	–0.061

25	Q8REA9	259	29,557.27	9.26	27,850	20.13	Stable	65.10	–0.547
26	Q8REB4	211	24,701.39	9.11	25,120	24.79	Stable	82.23	–0.678
27	Q8REB5	68	8,484.82	8.86	16,390	19.84	Stable	97.35	0.275
28	Q8REB6	89	10,424.15	5.12	15,595	20.20	Stable	107.30	–0.265
29	Q8REB7	90	9,741.08	4.81	2,980	46.19	Unstable	100.78	–0.031
30	Q8REB8	243	28,166.89	9.57	23,380	41.88	Unstable	97.41	–0.079
31	Q8REC0	116	13,843.31	9.02	16,960	28.95	Stable	102.33	–0.379
32	Q8REC7	300	33,893.21	5.51	23,965	26.54	Stable	83.20	–0.523
33	Q8REC8	243	29,136.48	8.45	22,475	54.01	Unstable	83.79	–0.886
34	Q8RED4	68	8,053.25	9.70	4,470	54.72	Unstable	78.82	–0.853
35	Q8RED5	62	7,661.08	9.82	5,960	34.60	Stable	95.81	–0.353
36	Q8REF3	109	12,279.30	7.74	11,920	37.42	Stable	100.09	0.038
37	Q8REG3	407	46,564.48	9.07	52,510	30.77	Stable	97.69	–0.162
38	Q8REH2	257	31,116.98	8.92	28,310	34.31	Stable	84.59	–0.516
39	Q8REI8	196	24,033.87	8.37	23,840	36.32	Stable	93.47	–0.559
40	Q8REK1	75	8,889.08	4.53	11,460	60.77	Unstable	101.33	–0.531
41	Q8REK2	48	5,684.76	9.16	-	31.43	Stable	95.42	–0.662
42	Q8REK5	120	14,068.33	5.26	5,960	27.96	Stable	94.08	–0.348
43	Q8REK8	304	33,766.84	7.68	17,545	26.77	Stable	114.80	0.405
44	Q8REL2	192	22,575.48	9.44	16,180	25.14	Stable	83.23	–0.588
45	Q8REM4	215	25,109.30	9.35	17,420	39.33	Stable	101.58	–0.272
46	Q8REM6	168	19,924.89	6.61	25,900	27.10	Stable	81.85	–0.441
47	Q8REN4	131	14,114.44	9.67	12950	23.92	Stable	150.38	1.213
48	Q8REN5	253	26,802.78	6.83	21,890	25.87	Stable	118.77	0.704
49	Q8REP0	255	30,564.36	9.21	35,870	32.07	Stable	113.22	0.157
50	Q8REP4	125	14,586.59	9.79	10,555	41.33	Unstable	117.68	0.422
51	Q8REP7	239	27,781.07	9.31	24,870	38.16	Stable	123.10	0.428
52	Q8REQ4	242	28,149.02	9.46	15,025	25.35	Stable	138.60	0.798
53	Q8RER4	179	20,159.34	9.46	14,900	31.58	Stable	134.97	0.874
54	Q8RES9	269	31,720.32	9.03	9,970	41.08	Unstable	57.47	–1.477
55	Q8RET2	114	13,426.38	8.67	28,420	11.16	Stable	86.32	–0.517
56	Q8RET3	179	20,218.90	9.52	13,410	28.15	Stable	105.08	0.142
57	Q8REU4	208	24,028.09	9.59	35,410	32.04	Stable	98.46	0.002
58	Q8REW7	247	29,960.28	8.93	37,945	43.33	Unstable	80.85	–0.735
59	Q8REX3	154	18,468.98	4.76	34,505	37.59	Stable	88.51	–0.458
60	Q8REX4	164	19,372.43	5.58	25,900	46.76	Unstable	98.11	–0.254
61	Q8REY7	270	29,549.72	7.02	42,400	29.11	Stable	83.44	–0.224
62	Q8REZ3	262	29,529.05	5.10	28,880	40.74	Unstable	77.79	–0.670
63	Q8REZ5	322	36,349.82	5.37	33,350	32.99	Stable	109.69	–0.054

64	Q8REZ8	167	19,502.58	9.63	17,420	33.30	Stable	129.46	0.278
65	Q8RF05	292	34,845.90	5.47	22,015	30.51	Stable	89.73	–0.411
66	Q8RF08	309	36,315.46	7.58	38,975	44.07	Unstable	117.99	–0.055
67	Q8RF26	127	14,533.97	9.37	5,960	25.97	Stable	91.34	–0.278
68	Q8RF27	154	18,835.25	4.86	17,420	41.27	Unstable	70.84	–1.112
69	Q8RF36	62	6,810.18	9.86	1,490	40.09	Unstable	122.74	0.411
70	Q8RF53	241	27,789.02	9.14	45,270	19.48	Stable	67.05	–0.687
71	Q8RF56	81	9,148.02	10.08	11,460	23.52	Stable	127.53	0.467
72	Q8RF58	85	9,906.97	4.72	8,940	12.40	Stable	64.24	–0.667
73	Q8RF72	69	8,076.25	4.69	2,980	56.90	Unstable	117025	–0.007
74	Q8RF74	66	7,691.73	7.89	12,950	-7.06	Stable	81.21	–0.612
75	Q8RF78	60	7,441.82	9.33	13,535	53.83	Unstable	110.50	0.255
76	Q8RF82	198	22,785.74	5.16	40,575	29.30	Stable	88.69	–0.357
77	Q8RF86	522	60,623.91	8.38	48,250	36.71	Stable	81.86	–0.718
78	Q8RF87	97	11,913.96	3.91	18,910	58.48	Unstable	68.35	–1.100
79	Q8RFB6	43	5,153.29	11.10	0	29.95	Stable	104.42	0.184
80	Q8RFD4	93	10,847.38	4.53	6,990	33.72	Stable	100.54	–0.522
81	Q8RFE5	270	33,013.48	9.86	38,280	34.24	Stable	99.67	–0.072
82	Q8RFF3	405	46,150.04	5.08	28,310	38.89	Stable	89.68	–0.618
83	Q8RFF4	430	47,163.40	7.77	33,810	26.78	Stable	119.07	0.737
84	Q8RFF9	156	18,197.21	9.56	10,430	36.74	Stable	100.58	–0.465
85	Q8RFH1	66	8,059.87	9.63	4,470	21.08	Stable	103.33	–0.124
86	Q8RFH2	99	11,789.86	9.02	8,940	39.20	Stable	108.38	–0.342
87	Q8RFH5	618	72,381.09	7.54	82,720	46.47	Unstable	105.50	–0.190
88	Q8RFH9	102	11,046.02	10.85	125	45.19	Unstable	71.67	–0.720
89	Q8RFI1	314	36,489.93	5.32	41,385	37.50	Stable	69.20	–0.778
90	Q8RFN1	134	15,242.99	6.12	7,700	55.69	Unstable	124.40	0.221
91	Q8RFN8	212	24,976.32	4.38	45,630	38.82	Stable	88.68	–0.521
92	Q8RFQ0	98	11,821.71	6.75	4,470	70.59	Unstable	105.51	–0.747
93	Q8RFQ3	119	14,093.28	9.14	29,910	26.99	Stable	84.29	–0.396
94	Q8RFR7	58	7,231.67	10.07	7,450	38.45	Stable	92.41	0.091
95	Q8RFS5	166	18,651.87	4.36	19,035	37.28	Stable	87.47	–0.462
96	Q8RFT5	236	26,939.77	4.88	12,950	42.53	Unstable	87.54	–0.443
97	Q8RFT7	166	19,137.96	5.11	7,450	35.30	Stable	113.80	–0.331
98	Q8RFU1	267	30,221.54	6.67	12,045	38.54	Stable	112.77	–0.036
99	Q8RFU6	77	9,012.70	9.84	4,470	45.20	Unstable	112.60	–0.318
100	Q8RFX3	206	23,765.11	4.68	21,555	43.80	Unstable	103.11	–0.375
101	Q8RFZ2	142	16,803.04	9.61	35,870	27.36	Stable	114.72	0.436
102	Q8RFZ5	34	4,124.03	9.04	2,980	46.84	Unstable	111.76	0.809

103	Q8RG13	66	7,500.81	9.22	4,470	49.00	Unstable	120.91	0.336
104	Q8RG23	73	8,529.72	4.76	11,460	53.28	Unstable	97.53	–0.495
105	Q8RG27	65	7,340.48	10.47	6,990	32.06	Stable	208.31	1.772
106	Q8RG66	164	19,514.36	5.32	16,515	26.73	Stable	82.01	–0.654
107	Q8RG68	56	6,908.93	4.79	8,480	27.56	Stable	90.36	–0.820
108	Q8RG71	12012	140,782.40	5.80	127,940	41.70	Unstable	104.86	–0.285
109	Q8RG81	107	13,012.12	9.30	12,950	55.24	Unstable	78.32	–0.631
110	Q8RG93	231	27,792.53	8.84	14,440	39.73	Stable	114.76	–0.533
111	Q8RG95	196	23,439.73	8.18	40,130	27.51	Stable	80.56	–0.704
112	Q8RGC0	772	90,124.20	8.99	161,525	27.02	Stable	85.73	0.049
113	Q8RGC2	295	35,413.88	4.94	40,925	55.26	Unstable	105.73	–0.313
114	Q8RGE9	139	16,060.76	6.72	25,565	18.80	Stable	117.05	0.340
115	Q8RGF5	224	26,890.26	7.58	23,380	33.94	Stable	103.17	–0.764
116	Q8RGF9	157	18,427.82	4.01	24,995	45.27	Unstable	98.09	–0.231
117	Q8RGG1	112	13,062.87	5.12	4,470	47.82	Unstable	101.79	–0.362
118	Q8RGK4	308	36,849.28	8.90	35,885	31.41	Stable	105.32	–0.182
119	Q8RGL1	427	48,724.26	8.92	47,220	22.96	Stable	87.45	–0.264
120	Q8RGM7	129	14,477.09	4.83	7,450	31.78	Stable	83.57	–0.587
121	Q8RGP8	310	35,958.73	5.17	29,590	34.69	Stable	82.97	–0.525
122	Q8RGQ3	318	36,440.73	9.01	48,945	32.51	Stable	109.78	0.131
123	Q8RGQ7	81	9,805.20	8.76	35,535	34.04	Stable	55.31	–0.670
124	Q8RGQ9	425	47,386.00	6.33	21,110	38.18	Stable	109.84	–0.136
125	Q8RGU2	400	48,278.42	8.36	78,855	55.14	Unstable	89.90	–0.531
126	Q8RGW1	121	14,200.57	9.52	5,960	41.00	Unstable	173.14	1.430
127	Q8RGY0	162	19,726.63	9.22	26,820	24.80	Stable	74.51	–0.637
128	Q8RGY2	85	10,157.66	4.50	7,450	46.44	Unstable	90.59	–0.395
129	Q8RGZ2	150	17,167.50	8.92	27,390	21.88	Stable	77.40	–0.629
130	Q8RGZ9	56	7,042.52	7.96	13,410	57.32	Unstable	158.39	0.959
131	Q8RH06	98	10,527.63	4.25	4,470	50.05	Unstable	149.18	1.314
132	Q8RH09	55	7,079.53	9.40	7,450	14.55	Stable	109.82	0.778
133	Q8RH22	74	8,949.81	9.36	7,450	26.64	Stable	132.97	1.477
134	Q8RH50	110	12,092.06	8.89	-	22.57	Stable	73.55	–0.834
135	Q8RH72	100	11,550.49	8.96	12,950	24.33	Stable	88.80	–0.576
136	Q8RH77	1607	187,720.79	6.60	230,970	36.02	Stable	95..88	–0.483
137	Q8RH78	329	38,628.28	8.85	44,155	27.24	Stable	83.53	–0.304
138	Q8RH83	42	5,017.14	8.59	4,595	47.01	Unstable	120.71	1.179
139	Q8RHC2	151	17,354.02	9.21	13,535	38.57	Stable	81.99	–0.538
140	Q8RHD8	125	14,301.66	10.09	14,565	34.70	Stable	129.36	0.5588
141	Q8RHE6	128	15,149.48	9.05	7,450	14.08	Stable	101.95	–0.289

142	Q8RHE9	389	46,509.82	4.64	38,865	53.88	Unstable	102.93	–0.410
143	Q8RHG1	309	35,935.61	5.54	39,225	37.94	Stable	81.72	–0.529
144	Q8RHG5	83	9,273.13	5.19	7,450	51.84	Unstable	140.84	1.092
145	Q8RHP4	70	8,421.63	7.91	5,960	10.69	Stable	69.71	–1.031
146	Q8RHQ2	175	19,277.28	8.31	20,525	25.26	Stable	98.17	–0.107
147	Q8RHR0	323	38,342.18	8.94	35,300	2741	Stable	71.80	–1.041
148	Q8RHR2	75	8,960.49	8.91	10,095	57.56	Unstable	91.07	–0.713
149	Q8RHR3	196	22,846.77	8.82	21,220	55.65	Unstable	107.50	–0.056
150	Q8RHR6	347	41,211.19	8.77	45,855	26.41	Stable	80.61	–0.807
151	Q8RHS6	600	71,383.33	8.35	70,265	31.55	Stable	94.25	–0.557
152	Q8RHS9	96	11,638.23	5.42	15,930	37.26	Stable	86.35	–0.598
153	Q8RHU8	109	12,251.21	6.57	9,970	24.98	Stable	116.15	–0.127
154	Q8RHW0	64	8,220.84	9.27	20,400	33.68	Stable	102.03	0.689
155	Q8RHW8	84	9,951.31	5.00	8,480	29.00	Stable	75.48	–1.157
156	Q8RHX5	320	35,614.22	5.79	15,025	22.67	Stable	108.69	–0.098
157	Q8RHY8	126	14,273.39	9.15	20,400	23.60	Stable	86.59	–0.496
158	Q8RHZ3	208	24,328.45	9.01	24,410	22.43	Stable	103.99	–0.195
159	Q8RI03	121	13,457.85	4.27	1,490	44.22	Unstable	89.34	–0.536
160	Q8RI09	279	32,819.06	9.44	58,790	45.25	Unstable	144.27	1.006
161	Q8RI11	145	15,092.37	8.73	10,095	8.21	Stable	99.38	0.083
162	Q8RI28	125	13,549.30	5.06	18,450	11.46	Stable	72.64	–0.389
163	Q8RI34	52	6,599.92	9.76	8,940	57.73	Unstable	125.58	0.45
164	Q8RI90	94	11,084.78	4.66	10,095	65.05	Unstable	106.70	–0.295
165	Q8RI98	88	10,569.45	4.81	9,970	29.54	Stable	109.66	–0.109
166	Q8RIA4	69	7,339.63	10.10	1,490	32.84	Stable	89.28	–0.278
167	Q8RIB1	328	38,614.88	8.57	38,865	42.03	Unstable	109.88	–0.298
168	Q8RIB2	193	22,866.74	8.45	32,890	32.49	Stable	98.86	–0.330
169	Q8RIC8	61	7,694.34	9.84	8,940	24.19	Stable	111.80	–0.072
170	Q8RID7	111	13,262.34	8.89	10,430	29.27	Stable	97.48	–0.612
171	Q8RID9	484	56,876.53	7.98	74,970	37.44	Stable	96.88	–0.443
172	Q8RII7	263	29,229.14	5.28	30,035	23.00	Stable	90.04	–0.303
173	Q8RIJ3	168	20,251.10	4.90	38,390	44.08	Unstable	103.27	–0.499
174	Q8RIJ4	57	6,774.68	4.99	5,960	41.15	Unstable	78.42	–0.649
175	Q8RIK2	53	6,639.17	9.82	8,940	77.91	Unstable	121.32	1.030
176	Q8RIK4	393	42,102.34	8.24	66,975	22.49	Stable	119.41	0.981
177	Q8RE79	214	26,079.46	9.48	33,475	29.80	Stable	93.27	–0.529
178	Q8RE80	365	42,828.27	9.58	64,415	29.83	Stable	123.64	0.533
179	Q8REC1	165	19,019.60	9.70	16,960	17.01	Stable	111.70	–0.179
180	Q8RED3	63	7,516.82	9.30	7,575	22.16	Stable	83.65	0.084

181	Q8REK4	414	49,117.92	4.96	46,885	47.92	Unstable	96.57	–0.592
182	Q8REK7	142	16,366.06	6.18	4,595	34.14	Stable	107.04	–0.312
183	Q8REQ2	245	28,208.31	9.73	45,965	37.49	Stable	111.02	0.423
184	Q8REQ3	269	30,609.12	7.72	27,850	46.88	Unstable	104.05	–0.223
185	Q8RET5	157	18,297.54	6.83	29,575	35.12	Stable	68.41	–0.787
186	Q8RF55	58	7,206.44	8.89	11,460	14.61	Stable	57.07	–0.779
187	Q8RF57	73	8,278.80	4.96	3,105	30.46	Stable	117.40	0.162
188	Q8RF73	54	6,670.14	7.76	8,605	23.07	Stable	128.15	0.981
189	Q8RF84	154	17,220.68	8.42	10,555	18.47	Stable	76.49	–0.349
190	Q8RF95	288	33,147.55	8.52	30,830	36.35	Stable	84.24	–0.692
191	Q8RFF2	243	28,330.02	8.99	23,505	48.20	Unstable	104.32	–0.324
192	Q8RFI2	290	32,354.48	5.44	17,880	24.89	Stable	98.52	0.078
193	Q8RFK6	153	16,965.94	4.67	14,440	32.25	Stable	74.51	–0.538
194	Q8RFM9	143	16,969.85	5.41	9,065	34.01	Stable	112.45	–0.216
195	Q8RFS4	143	16,263.07	9.16	15,930	25.83	Stable	98.11	–0.231
196	Q8RFV9	72	8,864.94	9.58	1,355	47.60	Unstable	157.08	1.021
197	Q8RFW1	163	20,165.45	9.45	30,370	29.9	Stable	131.47	0.826
198	Q8RG53	410	45,674.98	9.05	36,790	33.77	Stable	84.66	–0.559
199	Q8RGQ5	37	4,270.90	3.95	8,480	41.88	Unstable	86.76	0.084
200	Q8RGU3	143	17,119.27	4.99	14,900	40.24	Unstable	140.35	0.447
201	Q8RGW8	305	36,580.68	8.85	38,070	35.94	Stable	92.66	–0.579
202	Q8RGX2	413	49,860.15	7.51	109,670	43.08	Unstable	71.74	–1.081
203	Q8RH27	111	12,568.58	7.65	6,085	35.90	Stable	98.29	–0.345
204	Q8RH75	220	25,801.42	8.65	29,800	28.34	Stable	83.68	–0.410
205	Q8RH79	271	31,801.16	5.64	44,810	38.02	Stable	85.28	–0.625
206	Q8RHC4	64	7,687.94	9.33	11,920	21.15	Stable	88.28	–0.770
207	Q8RHE7	189	22,226.84	8.32	23,505	45.17	Unstable	101.53	–0.203
208	Q8RHK2	257	30,649.19	8.22	53,180	24.78	Stable	64.44	–1.172
209	Q8RHL9	237	28,048.47	5.79	22,140	33.19	Stable	99.41	–0.316
210	Q8RHR1	155	17,811.03	9.56	13,075	39.42	Stable	113.16	0.139
211	Q8RHT3	1175	133,515.19	8.99	90,430	24.04	Stable	94.80	–0.522
212	Q8RHV0	60	6,904.21	6.16	2,980	81.83	Unstable	105.50	0.210
213	Q8RHV7	91	10,133.41	5.44	14,105	15.50	Stable	77.03	–0.370
214	Q8RHX0	242	29,249.71	9.53	43,905	29.12	Stable	113.14	0.108
215	Q8RI20	233	28,494.05	8.75	60,850	47.27	Unstable	79.91	–0.723
216	Q8RI29	264	28,915.17	8.54	27,960	14.02	Stable	86.74	–0.183
217	Q8RI81	265	30,238.69	5.33	26,610	31.18	Stable	89.02	–0.281
218	Q8RIF3	139	15,490.87	8.81	13,075	34.18	Stable	87.63	–0.040
219	Q8RIP2	123	14,185.37	5.42	4,470	26.77	Stable	102.36	–0.400

220	Q8RIP4	129	14,764.93	5.39	4,470	34.77	Stable	86.20	–0.747
221	Q8RIR0	25	2,982.64	7.98	1,490	60.16	Unstable	136.40	1.408
222	Q8RGD0	255	29,699.60	5.53	27,850	37.24	Stable	85.22	–0.573
223	Q8RFW3	179	21,819.87	5.03	15,025	23.90	Stable	92.57	–0.754
224	Q8RI22	192	22,377.50	4.90	22,920	31.56	Stable	92.92	–0.393
225	Q8RGU6	114	12,737.04	8.59	3,230	32.77	Stable	94.65	–0.193
226	Q8REU5	241	28,295.08	9.31	38,850	18.30	Stable	93.44	–0.004
227	Q8RF11	322	37,964.79	5.28	23,965	32.40	Stable	99.29	–0.321
228	Q8RF13	243	27,662.59	8.81	19,495	26.75	Stable	93.46	–0.248
229	Q8RDP5	94	11,242.77	4.85	20,650	54.89	Unstable	79.79	–0.298
230	Q8RDT0	79	9,406.40	10.15	7,450	24.74	Stable	118.35	0.513
231	Q8REV8	85	9,605.72	10.15	5,500	24.56	Stable	147.88	0.995
232	Q8REV9	122	13,909.80	5.14	13,535	47.45	Unstable	90.33	–0.285
233	Q8RHF3	196	23,169.61	4.96	51,005	23.30	Stable	97.96	–0.319
234	Q8RET4	294	35,206.95	9.71	43,320	37.20	Stable	120.99	0.115
235	Q8RF10	187	22,401.56	9.28	20,400	29.57	Stable	112.57	0.006
236	Q8REC4	299	35,696.76	9.82	41,050	33.52	Stable	90.64	–0.360
237	Q8RFA9	251	30,374.63	9.04	47,580	28.83	Stable	95.46	–0.299
238	Q8RG02	205	24,149.68	9.53	29,130	29.85	Stable	100.73	0.227
239	Q8RHW9	84	9,745.08	4.99	6,990	40.89	Unstable	65.12	–1.002
240	Q8RFQ1	119	14,195.71	8.60	7,450	50.64	Unstable	103.95	–0.473
241	Q8RHE1	65	7,645.02	6.12	5,960	34.95	Stable	136.31	0.269
242	Q8RF54	112	13,139.86	9.83	20,065	57.13	Unstable	106.25	–0.026
243	Q8RGZ1	103	12,395.57	6.33	9,065	52.60	Unstable	89.90	–0.671
244	Q8RDZ6	144	17,114.61	9.23	16,765	38.36	Stable	119.79	1.167
245	Q8RHB0	90	10,652.60	8.80	9,970	20.82	Stable	121.33	–0.088
246	Q8RHP6	82	9,438.48	4.28	7,450	30.18	Stable	86.83	–0.546
247	Q8RH10	91	10,725.22	4.79	14,440	56.65	Unstable	83.63	–0.665
248	Q8RID5	117	13,920.44	9.42	8,940	33.36	Stable	110.77	–0.212
249	Q8RGA5	81	9,876.77	9.68	4,470	44.38	Unstable	97.41	–0.572
250	Q8RIP3	118	13,813.41	4.61	5,960	50.12	Unstable	65.42	–1.269
251	Q8RGV4	461	53,021.16	5.09	29,340	30.75	Stable	100.59	–0.479
252	Q8RGK6	80	9,180.63	4.73	13,980	13.82	Stable	107.12	–0.096
253	Q8RH61	162	18,554.29	4.54	4,470	46.47	Unstable	59.20	–1.194
254	Q8RI31	388	41,761.36	6.02	28,085	37.37	Stable	135.44	0.978
255	Q8RII9	54	6,272.50	9.03	1,490	48.92	Unstable	133.70	0.426

256	Q8RI97	326	38,864.40	4.81	41,385	41.24	Unstable	101.63	–0.297
257	Q8RIE1	236	27,812.88	6.95	15,150	39.75	Stable	123.90	0.650
258	Q8RF09	78	8,863.22	7.90	2,980	65.61	Unstable	106.28	–0.574
259	Q8RHP7	124	14,800.86	4.94	11,920	35.71	Stable	102.98	-0.443
260	Q8RE00	69	8,651.56	9.90	14,440	23.47	Stable	101.59	0.578
261	Q8RGS2	180	21,351.10	8.78	12,950	35.10	Stable	92.06	–0.436
262	Q8REL7	91	10,571.94	9.94	11,460	25.65	Stable	148.79	0.968
263	Q8RI92	239	27,855.95	9.07	45,380	34.26	Stable	81.55	–0.589
264	Q8REX6	226	26,700.35	8.65	32,320	34.92	Stable	82.39	–0.681
265	Q8RHH4	218	25,416.07	5.36	21,890	48.17	Unstable	93.94	–0.583
266	Q8RFC5	139	16,300.83	8.76	13,535	30.52	Stable	94.03	–0.213
267	Q8RGW0	76	9,283.29	9.26	15,595	34.49	Stable	107.76	0.511
268	Q8RE40	56	7,186.77	9.87	10,430	26.54	Stable	109.64	0.814
269	Q8RIC0	24	2,812.37	10.22	-	-2.58	Stable	69.17	–1.496
270	Q8RFS7	1155	133,557.41	8.07	183,710	25.98	Stable	62.71	–0.835
271	Q8REL5	85	9,825.14	4.23	11,460	14.60	Stable	95.29	–0.135
272	Q8RFJ9	50	6,068.74	4.29	15,470	30.47	Stable	46.80	–0.608
273	Q8REZ1	177	19,010.55	9.50	2,980	220.5	Stable	104.24	0.053
274	Q8REL8	69	8,329.67	5.55	2,980	24.19	Stable	93.19	–1.129
275	Q8RGC3	282	33,052.76	7.11	51,800	38.42	Stable	82.30	–0.594
276	Q8RES6	74	8,518.26	9.92	1,490	17.45	Stable	117.16	0.616
277	Q8RGV8	135	16,506.79	4.91	29,910	37.58	Stable	96.07	-0.624
278	Q8RF79	133	15,539.23	3.99	15,930	71.81	Unstable	91.65	–0.380
279	Q8REE9	104	12,513.79	9.14	9,970	58.73	Unstable	103.94	–0.662
280	Q8RFG7	177	20,359.14	8.80	21,890	35.52	Stable	81.58	–0.808
281	Q8RF46	257	29,677.17	9.21	43,445	31.09	Stable	79.30	–0.484
282	Q8RFV6	57	6,768.81	4.65	2,980	48.14	Unstable	75.26	–0.677
283	Q8RFI6	225	27,591.63	8.29	26,360	45.94	Unstable	81.02	–0.867
284	Q8RG54	110	13,204.90	5.27	8,940	43.75	Unstable	87.73	–0.709
285	Q8RFL2	81	9,112.63	5.46	3,355	64.44	Unstable	45.80	–0.193
286	Q8RE95	151	18,262.89	5.26	27,390	31.25	Stable	95.56	–0.493
287	Q8RIQ4	64	7,223.84	9.47	4,595	15.72	Stable	117.34	0.941

288	Q8RIL7	167	18,946.79	4.69	13,410	63.07	Unstable	70.78	–0.987
289	Q8RFW7	183	22,110.56	8.61	16,515	17.47	Stable	90.49	–0.697
290	Q8RFG4	109	12,969.95	5.68	17,210	11.42	Stable	87.61	–0.318
291	Q8RHV3	58	6,955.22	8.93	1,490	23.14	Stable	87.24	–0.774
292	Q8REB2	213	25,389.10	8.96	31,650	40.34	Unstable	73.24	–0.952
293	Q8RH59	48	5,776.96	6.01	4,470	53.30	Unstable	150.21	0.598
294	Q8REP2	199	23,644.13	9.47	26,945	30.6	Stable	112.71	0.210
295	Q8RFJ2	64	7,157.84	9.82	17,990	34.55	Stable	140.00	0.998
296	Q8REB3	177	21,359.74	8.84	20,525	40.67	Unstable	103.56	–0.486
297	Q8RI49	151	17,126.82	9.13	21,890	22.51	Stable	90.33	–0.393
298	Q8RI95	189	22,161.85	7.62	37,485	26.73	Stable	95.82	–0.232
299	Q8RER1	212	24,454.53	9.04	26,930	25.33	Stable	110.33	–0.105
300	Q8RGC4	303	35,128.50	9.04	50,880	39.54	Stable	81.42	–0.238
301	Q8RF59	169	19,943.78	5.02	25,330	39.52	Stable	100.24	–0.451
302	Q8REJ6	165	19,085.96	5.30	17,420	29.97	Stable	105.64	–0.135
303	Q8REX8	82	9,828.13	6.09	27,055	14.32	Stable	62.93	–0.634
304	Q8RGB9	306	36,213.66	9.06	70,710	31.54	Stable	63.40	–0.805
305	Q8RE01	397	46,331.25	6.01	44,725	27.83	Stable	86.42	–0.327
306	Q8RIC3	58	7,211.76	9.90	8,940	21.54	Stable	122.59	0.060
307	Q8RF19	101	11,679.17	8.73	5,960	45.50	Unstable	81.98	–0.710
308	Q8RI48	157	18,243.83	5.97	13,535	41.07	Unstable	86.24	–0.422
309	Q8RHE0	134	16,058.55	9.95	18,910	27.71	Stable	76.42	–0.846
310	Q8RF83	539	64,667.01	4.86	90,540	37.62	Stable	84.82	–0.751
311	Q8RE50	131	15,359.85	4.99	12,950	33.03	Stable	110.76	–0.250
312	Q8RGV9	55	6,446.31	6.17	2,980	23.73	Stable	72.55	–0.918
313	Q8RDP4	184	22,269.55	5.87	29,630	33.65	Stable	90.05	–0.548
314	Q8RHL3	186	21,718.88	5.44	19,370	34.40	Stable	98.49	–0.412
315	Q8RH94	182	21,511.15	4.53	20,985	44.91	Unstable	69.67	–0.514
316	Q8RGW2	75	8,962.95	3.98	8,605	66.14	Unstable	81.87	–0.560
317	Q8RFW6	137	16,601.47	5.64	19,370	32.44	Stable	93.87	0.464
318	Q8RFK7	153	17,373.91	4.97	11,460	32.16	Stable	79.54	–0.444
319	Q8RDN2	188	22,606.93	8.75	27,850	19.41	Stable	92.18	–0.521

320	Q8RHW2	99	11,686.18	4.89	7,450	28.57	Stable	86.57	–0.613
321	Q8RE27	72	8,140.68	6.82	5,625	44.46	Unstable	120.42	0.183
322	Q8RIK7	456	51,473.92	5.34	103,960	24.48	Stable	72.46	–0.586
323	Q8RFM6	205	24,352.51	5.34	27,390	42.65	Unstable	72.20	–0.897
324	Q8RG60	141	16,898.78	9.79	41,035	30.18	Stable	142.41	0.972
325	Q8RE17	60	7,226.73	9.59	6,085	38.02	Stable	102.33	0.165
326	Q8RFH0	211	24,133.70	4.12	33,725	24.51	Stable	72.56	–0.500
327	Q8REM3	297	34,617.34	9.56	20,650	18.50	Stable	154.58	0.801
328	Q8RIB4	262	31,609.39	9.10	45,395	29.70	Stable	79.20	–0.615
329	Q8RFE3	118	13,249.06	5.21	4,970	43.56	Unstable	76.02	–0.355
330	Q8RDX6	201	23,928.28	4.59	21,890	46.85	Unstable	105.17	–0.266
331	Q8REK3	595	69,040.83	8.52	33,935	28.67	Stable	92.08	–0.631
332	Q8RGY3	160	18,995.69	4.75	16,390	38.02	Stable	101.06	–0.404
333	Q8RH29	195	23,229.80	6.10	20,860	40.69	Unstable	102.97	–0.618
334	Q8RG37	132	15,441.22	9.15	7,575	47.14	Unstable	101.29	–0.320
335	Q8RH93	218	26,328.07	5.85	44,475	40.92	Unstable	87.66	–0.610
336	Q8REV5	256	30,392.25	4.83	43,570	33.36	Stable	75.74	–0.626
337	Q8RFB9	137	15,603.06	5.27	9,970	22.07	Stable	108.91	–0.266
338	Q8REP9	108	12,524.13	4.51	11,920	22.81	Stable	89.44	–0.519
339	Q8RGQ6	232	27,907.10	7.64	58,790	35.87	Stable	79.78	–0.506
340	Q8REQ5	43	4,950.51	4.32	15,470	55.60	Unstable	86.28	–0.388
341	Q8RHN1	59	7,063.44	9.48	7,450	26.47	Stable	128.81	0.124
342	Q8RHE8	179	21,397.71	8.54	21,890	30.81	Stable	96.93	–0.399
343	Q8REI4	109	12,305.25	6.58	5,960	34.59	Stable	97.52	–0.221
344	Q8REN7	184	21,673.95	9.63	25,900	42.45	Unstable	117.66	0.738
345	Q8RGA1	174	19,158.82	5.66	12,950	37.37	Stable	90.34	–0.176
346	Q8RG08	133	14,490.03	4.79	18,575	12.76	Stable	115.04	0.829
347	Q8RFA5	317	37,424.26	4.97	51,925	32.17	Stable	79.87	–0.463
348	Q8RG74	57	6,601.15	9.40	5,960	17.71	Stable	145.26	1.126
349	Q8REC6	517	61,460.88	6.18	61,785	29.43	Stable	86.36	–0.689
350	Q8REX1	153	18,338.88	5.31	33,350	52.67	Unstable	86.67	–0.552
351	Q8RGC5	295	34,879.22	8.93	22,475	41.60	Unstable	109.69	0.056

352	Q8RI91	242	28,893.80	8.25	26,820	32.95	Stable	112.77	–0.248
353	Q8RF75	77	8,509.15	9.80	2,980	34.71	Stable	138.05	0.387
354	Q8RH91	156	18,100.96	7.66	9,065	43.94	Unstable	91.22	–0.209
355	Q8RGY1	62	7,385.97	9.18	6,085	42.70	Unstable	149.35	1.224
356	Q8RGH9	151	17,779.44	9.15	21,890	36.11	Stable	90.26	–0.734
357	Q8RHH0	167	19,878.80	6.85	24,410	31.35	Stable	92.81	–0.439
358	Q8REW6	258	31,352.77	8.90	46,300	36.83	Stable	78.14	–0.700
359	Q8RH76	216	25,191.56	7.65	28,310	28.78	Stable	81.67	–0.517
360	Q8RH01	57	6,631.11	10.13	5,960	5.21	Stable	99.30	0.626
361	Q8RHR5	387	46,777.38	9.54	53,875	37.09	Stable	126.15	0.242
362	Q8RH12	158	18,674.76	10.10	17,420	33.09	Stable	142.41	0.619
363	Q8RGE8	144	16,861.38	9.10	21,890	32.74	Stable	65.69	–0.374
364	Q8RE96	200	22,890.38	9.28	13,980	31.96	Stable	133.05	0.261
365	Q8RGY4	20	2,542.07	8.10	5,960	45.57	Unstable	112.00	0.475
366	Q8RFW4	65	7,597.76	8.14	6,990	40.22	Unstable	186.00	1.685
367	Q8RIH9	179	20,664.85	5.39	13,535	31.42	Stable	107.71	–0.176
368	Q8RI40	79	9,742.06	4.74	15,930	46.72	Unstable	88.86	–0.916
369	Q8RFK8	467	55,197.44	9.16	63,635	34.39	Stable	93.49	–0.396
370	Q8RHC5	164	19,067.60	8.45	24,870	45.39	Unstable	85.00	–0.606
371	Q8RIC7	34	3,958.87	9.66	4,470	90.65	Unstable	94.71	0.141
372	Q8RIM4	177	20,338.14	6.31	15,025	31.28	Stable	129.49	0.501
373	Q8RG38	101	11,294.96	8.96	5,960	51.83	Unstable	90.59	–0.197
374	Q8RH73	150	17,598.54	8.53	27,390	25.89	Stable	115.67	0.003
375	Q8RF03	219	25,821.03	9.09	33,015	31.39	Stable	129.04	0.384
376	Q8RHP8	64	7,821.76	8.53	9,065	39.86	Stable	167.34	1.828
377	Q8RGG0	114	12,784.83	4.35	6,085	32.00	Stable	102.63	0.428
378	Q8RHC3	65	7,432.65	5.36	2,980	16.10	Stable	97.54	–0.298
379	Q8RFH7	239	27,956.79	5.22	36,900	31.25	Stable	88.49	–0.505
380	Q8RHV9	59	6,686.75	5.26	2,115	30.71	Stable	42.88	–0.524
381	Q8RH69	62	7,683.84	5.82	5,960	42.43	Unstable	81.77	–1.055
382	Q8RGP0	54	6,511.51	5.71	10,430	31.97	Stable	85.00	0.259
383	Q8RGP7	62	7,383.18	9.52	4,470	14.02	Stable	155.65	1.387

384	Q8RDV5	99	11,600.26	4.69	2,980	33.69	Stable	96.46	–0.262
385	Q8RE05	138	15,756.42	4.97	8,940	43.84	Unstable	116.45	0.002
386	Q8RGX9	128	15,632.29	9.87	17,880	35.85	Stable	155.31	0.929
387	Q8RGQ2	147	17,198.40	4.99	15,930	44.65	Unstable	83.54	–0.699
388	Q8RF70	204	24,564.83	4.77	37,360	48.87	Unstable	76.96	–0.855
389	Q8RI41	279	33,263.49	4.93	77,935	42.46	Unstable	82.51	–0.491
390	Q8RDW0	578	69,475.62	5.47	83,215	24.60	Stable	86.64	–0.535
391	Q8RF29	140	16,123.71	6.58	7,700	36.29	Stable	114.93	–0.159
392	Q8RIA8	184	21,384.47	8.47	15,150	29.15	Stable	85.33	–0.699
393	Q8R6H9	82	95,85.95	5.40	12,950	52.39	Unstable	98.66	–0.651
394	Q8R6K0	119	14,445.40	5.29	14,440	50.27	Unstable	81.09	–1.155
395	Q8R6I0	26	3,426.12	9.30	8,480	31.36	Stable	101.15	0.254
396	Q8R6K1	52	6,709.01	8.76	18,910	44.51	Unstable	105.00	0.712
397	Q8RHM0	144	17,116.12	9.96	15,930	32.09	Stable	61.60	–0.643
398	Q8RHF0	226	26,625.59	5.39	11,920	50.40	Unstable	63.81	–1.299
