# Supplementary material for: Functional annotation of uncharacterized proteins from Fusobacterium nucleatum: identification of virulence factors
Source: Genomics Inform. 2023 Jun 30;21(2):e21. doi: 10.5808/gi.22065 (PMC10326533; doi:10.5808/gi.22065)
Supplement: Supplementary Table 4. — Predicted sub-cellular localization (Cello server), signal peptide (SignalP 5.0) and transmembrane helices (TMHMM) for the list of Fusobacterium nucleatum uncharacterized proteins of F. nucleatum [file gi-22065-Supplementary-Table-4.pdf]

Supplementary Table 4. Predicted sub-cellular localization (Cello server), signal peptide (SignalP 5.0) and transmembrane helices (TMHMM) for the list of *Fusobacterium nucleatum* uncharacterized proteins of *F. nucleatum*

S. No.	Accession ID	Localization	Signal peptide	Transmembrane helices (TMHMM)
1	Q8R6J3	Cytoplasm	Signal peptide Sec/SPI (0.9978)	0
2	Q8R669	Cytoplasm	-	0
3	Q8RDL8	Cytoplasm	Signal peptide (Sec/SPI) 0.4912	1
4	Q8RDM0	Cytoplasm	Signal peptide (Sec/SPI) 0.6626	0
5	Q8RDN3	Periplasm	Lipoprotein signal peptide (Sec/SPII) 0.9751	0
6	Q8RDP1	Outer membrane	-	0
7	Q8RDR8	Cytoplasm	-	0
8	Q8RDV4	Cytoplasm	-	1
9	Q8RDW3	Outer membrane	-	0
10	Q8RDY5	Cytoplasm	-	1
11	Q8RE02	Cytoplasm	-	0
12	Q8RE16	Cytoplasm	-	1
13	Q8RE33	Outer membrane	-	0
14	Q8RE35	Outer membrane	-	1
15	Q8RE36	Outer membrane	-	1
16	Q8RE37	Extracellular	-	1
17	Q8RE38	Cytoplasm	-	1
18	Q8RE39	Outer membrane	-	2
19	Q8RE61	Cytoplasm	-	0
20	Q8RE69	Periplasm	-	0
21	Q8RE90	Cytoplasm	-	0

22	Q8RE93	Cytoplasm	-	0
23	Q8REA1	Inner membrane	-	6
24	Q8REA8	Cytoplasm	-	2
25	Q8REA9	Outer membrane	Signal peptide (Sec/SPI) 0.488	0
26	Q8REB4	Cytoplasm	-	0
27	Q8REB5	Periplasm	-	0
28	Q8REB6	Cytoplasm	-	0
29	Q8REB7	Cytoplasm	-	1
30	Q8REB8	Outer membrane	-	0
31	Q8REC0	Cytoplasm	-	0
32	Q8REC7	Outer membrane	Signal peptide (Sec/SPI) 0.547	0
33	Q8REC8	Cytoplasm	-	0
34	Q8RED4	Cytoplasm	-	0
35	Q8RED5	Cytoplasm	-	0
36	Q8REF3	Periplasm	Lipoprotein signal peptide (Sec/SPII) 0.8064	0
37	Q8REG3	Cytoplasm	-	0
38	Q8REH2	Cytoplasm	-	0
39	Q8REI8	Cytoplasm	-	1
40	Q8REK1	Cytoplasm	-	0
41	Q8REK2	Cytoplasm	-	0
42	Q8REK5	Cytoplasm	-	1
43	Q8REK8	Inner membrane	-	4
44	Q8REL2	Cytoplasm	-	0
45	Q8REM4	Cytoplasm	-	0
46	Q8REM6	Periplasm	-	1

47	Q8REN4	Inner membrane	-	4
48	Q8REN5	Inner membrane	-	7
49	Q8REP0	Inner membrane	-	3
50	Q8REP4	Inner membrane	-	1
51	Q8REP7	Inner membrane	-	4
52	Q8REQ4	Inner membrane	-	6
53	Q8RER4	Inner membrane	-	4
54	Q8RES9	Cytoplasm	-	1
55	Q8RET2	Cytoplasm	-	0
56	Q8RET3	Cytoplasm	-	2
57	Q8REU4	Inner membrane	-	4
58	Q8REW7	Cytoplasm	-	0
59	Q8REX3	Cytoplasm	-	1
60	Q8REX4	Cytoplasm	-	0
61	Q8REY7	Periplasm	Signal peptide (Sec/SPI) 0.9894	0
62	Q8REZ3	Cytoplasm	Other	0
63	Q8REZ5	Cytoplasm	-	1
64	Q8REZ8	Cytoplasm	-	2
65	Q8RF05	Cytoplasm	-	0
66	Q8RF08	Cytoplasm	-	0
67	Q8RF26	Cytoplasm	-	0
68	Q8RF27	Cytoplasm	-	0
69	Q8RF36	Cytoplasm	-	0
70	Q8RF53	Outer membrane	-	0
71	Q8RF56	Cytoplasm	-	2
72	Q8RF58	Cytoplasm	-	0

73	Q8RF72	Cytoplasm	-	0
74	Q8RF74	Cytoplasm	-	0
75	Q8RF78	Cytoplasm	-	1
76	Q8RF82	Cytoplasm	-	0
77	Q8RF86	Cytoplasm	-	0
78	Q8RF87	Cytoplasm	Signal peptide (Sec/SPI) 0.7626	0
79	Q8RFB6	Cytoplasm	-	0
80	Q8RFD4	Cytoplasm	-	0
81	Q8RFE5	Inner membrane	-	4
82	Q8RFF3	Outer membrane	-	1
83	Q8RFF4	Inner membrane	-	11
84	Q8RFF9	Cytoplasm	-	0
85	Q8RFH1	Cytoplasm	-	0
86	Q8RFH2	Cytoplasm	-	0
87	Q8RFH5	Cytoplasm	-	3
88	Q8RFH9	Periplasm	Lipoprotein signal peptide (Sec/SPII) 0.9842	1
89	Q8RFI1	Extracellular	Signal peptide (Sec/SPI) 0.8738	1
90	Q8RFN1	Cytoplasm	-	0
91	Q8RFN8	Cytoplasm	-	0
92	Q8RFQ0	Cytoplasm	-	0
93	Q8RFQ3	Cytoplasm	-	0
94	Q8RFR7	Cytoplasm	-	0
95	Q8RFS5	Cytoplasm	Lipoprotein signal peptide (Sec/SPII) 0.8115	0
96	Q8RFT5	Cytoplasm	-	0

97	Q8RFT7	Cytoplasm	-	0
98	Q8RFU1	Cytoplasm	-	0
99	Q8RFU6	Cytoplasm	-	0
100	Q8RFX3	Cytoplasm	Lipoprotein signal peptide (Sec/SPII) 0.9978	0
101	Q8RFZ2	Inner membrane	-	3
102	Q8RFZ5	Cytoplasm	-	2
103	Q8RG13	Periplasm	-	0
104	Q8RG23	Cytoplasm	-	0
105	Q8RG27	Inner membrane	-	2
106	Q8RG66	Cytoplasm	-	0
107	Q8RG68	Cytoplasm	-	0
108	Q8RG71	Cytoplasm	-	1
109	Q8RG81	Cytoplasm	-	0
110	Q8RG93	Cytoplasm	-	0
111	Q8RG95	Cytoplasm	-	0
112	Q8RGC0	Outer membrane	-	13
113	Q8RGC2	Cytoplasm	-	2
114	Q8RGE9	Inner membrane	-	2
115	Q8RGF5	Cytoplasm	-	0
116	Q8RGF9	Cytoplasm	-	0
117	Q8RGG1	Cytoplasm	-	0
118	Q8RGK4	Cytoplasm	-	2
119	Q8RGL1	Periplasm	Signal peptide (Sec/SPI) 0.8075	0
120	Q8RGM7	Periplasm	Signal peptide (Sec/SPI) 0.998	0
121	Q8RGP8	Cytoplasm	-	0

122	Q8RGQ3	Cytoplasm	-	4
123	Q8RGQ7	Cytoplasm	-	1
124	Q8RGQ9	Cytoplasm	-	0
125	Q8RGU2	Cytoplasm	-	0
126	Q8RGW1	Inner membrane	-	4
127	Q8RGY0	Cytoplasm	-	1
128	Q8RGY2	Cytoplasm	-	0
129	Q8RGZ2	Cytoplasm	-	0
130	Q8RGZ9	Cytoplasm	-	1
131	Q8RH06	Inner membrane	-	3
132	Q8RH09	Cytoplasm	-	1
133	Q8RH22	Inner membrane	-	2
134	Q8RH50	Cytoplasm	Signal peptide (Sec/SPI) 0.5774	0
135	Q8RH72	Cytoplasm	-	0
136	Q8RH77	Cytoplasm	-	0
137	Q8RH78	Cytoplasm	-	1
138	Q8RH83	Cytoplasm	-	1
139	Q8RHC2	Cytoplasm	-	0
140	Q8RHD8	Inner membrane	-	2
141	Q8RHE6	Cytoplasm	-	1
142	Q8RHE9	Cytoplasm	-	1
143	Q8RHG1	Cytoplasm	-	0
144	Q8RHG5	Cytoplasm	-	2
145	Q8RHP4	Cytoplasm	-	0
146	Q8RHQ2	Cytoplasm	-	0
147	Q8RHR0	Outer membrane	-	0

148	Q8RHR2	Cytoplasm	-	0
149	Q8RHR3	Cytoplasm	-	0
150	Q8RHR6	Cytoplasm	Lipoprotein signal peptide (Sec/SPII) 0.6496	0
151	Q8RHS6	Cytoplasm	-	0
152	Q8RHS9	Cytoplasm	-	0
153	Q8RHU8	Cytoplasm	-	1
154	Q8RHW0	Cytoplasm	-	1
155	Q8RHW8	Cytoplasm	-	0
156	Q8RHX5	Cytoplasm	-	0
157	Q8RHY8	Periplasm	-	0
158	Q8RHZ3	Cytoplasm	-	1
159	Q8RI03	Cytoplasm	Signal peptide (Sec/SPI) 0.9416	0
160	Q8RI09	Inner membrane	-	7
161	Q8RI11	Periplasm	Signal peptide (Sec/SPI) 0.8457	0
162	Q8RI28	Cytoplasm	-	0
163	Q8RI34	Cytoplasm	-	1
164	Q8RI90	Cytoplasm	-	0
165	Q8RI98	Cytoplasm	-	1
166	Q8RIA4	Periplasm	-	0
167	Q8RIB1	Cytoplasm	-	2
168	Q8RIB2	Cytoplasm	-	0
169	Q8RIC8	Cytoplasm	-	0
170	Q8RID7	Cytoplasm	-	0
171	Q8RID9	Cytoplasm	-	0
172	Q8RII7	Cytoplasm	-	0

173	Q8RIJ3	Cytoplasm	-	0
174	Q8RIJ4	Cytoplasm	-	0
175	Q8RIK2	Cytoplasm	-	1
176	Q8RIK4	Inner membrane	-	16
177	Q8RE79	Cytoplasm	-	0
178	Q8RE80	Inner membrane	-	11
179	Q8REC1	Cytoplasm	-	0
180	Q8RED3	Cytoplasm	-	0
181	Q8REK4	Cytoplasm	-	0
182	Q8REK7	Cytoplasm	-	0
183	Q8REQ2	Inner membrane	-	4
184	Q8REQ3	Cytoplasm	-	0
185	Q8RET5	Cytoplasm	-	0
186	Q8RF55	Cytoplasm	-	0
187	Q8RF57	Cytoplasm	Lipoprotein signal peptide (Sec/SPII) 0.7281	0
188	Q8RF73	Cytoplasm	-	2
189	Q8RF84	Cytoplasm	-	0
190	Q8RF95	Outer membrane	-	0
191	Q8RFF2	Cytoplasm	Lipoprotein signal peptide (Sec/SPII) 0.835	1
192	Q8RFI2	Cytoplasm	-	4
193	Q8RFK6	Extracellular	-	0
194	Q8RFM9	Cytoplasm	-	0
195	Q8RFS4	Cytoplasm	Signal peptide (Sec/SPI) 0.9428	1
196	Q8RFV9	Cytoplasm	-	2

197	Q8RFW1	Inner membrane	-	4
198	Q8RG53	Outer membrane	-	0
199	Q8RGQ5	Cytoplasm	-	0
200	Q8RGU3	Inner membrane	-	3
201	Q8RGW8	Cytoplasm	-	0
202	Q8RGX2	Outer membrane	Signal peptide (Sec/SPI) 0.8216	0
203	Q8RH27	Cytoplasm	-	0
204	Q8RH75	Periplasm	Signal peptide (Sec/SPI) 0.6131	0
205	Q8RH79	Cytoplasm	-	0
206	Q8RHC4	Cytoplasm	-	0
207	Q8RHE7	Cytoplasm	-	0
208	Q8RHK2	Outer membrane	-	0
209	Q8RHL9	Cytoplasm	-	0
210	Q8RHR1	Cytoplasm	Signal peptide (Sec/SPI) 0.5055	2
211	Q8RHT3	Outer membrane	-	0
212	Q8RHV0	Cytoplasm	-	0
213	Q8RHV7	Cytoplasm	-	0
214	Q8RHX0	Inner membrane	-	4
215	Q8RI20	Cytoplasm	-	0
216	Q8RI29	Periplasm	Signal peptide (Sec/SPI) 0.9546	0
217	Q8RI81	Cytoplasm	-	0
218	Q8RIF3	Periplasm	-	0
219	Q8RIP2	Cytoplasm	Signal peptide (Sec/SPI) 0.9634	0
220	Q8RIP4	Cytoplasm	Lipoprotein signal peptide (Sec/SPII) 0.9979	0
221	Q8RIR0	Cytoplasm	-	1

222	Q8RGD0	Outer membrane	Signal peptide (Sec/SPI) 0.6769	1
223	Q8RFW3	Cytoplasm	Other	0
224	Q8RI22	Cytoplasm	Other	0
225	Q8RGU6	Cytoplasm	Other	0
226	Q8REU5	Inner membrane	Other	2
227	Q8RF11	Cytoplasm	Other	0
228	Q8RF13	Outer membrane	Signal peptide (Sec/SPI) 0.8937	1
229	Q8RDP5	Cytoplasm	Other	0
230	Q8RDT0	Cytoplasm	Other	2
231	Q8REV8	Inner membrane	Other	2
232	Q8REV9	Cytoplasm	Other	0
233	Q8RHF3	Cytoplasm	Other	0
234	Q8RET4	Inner membrane	Other	4
235	Q8RF10	Inner membrane	Other	2
236	Q8REC4	Outer membrane	Other	0
237	Q8RFA9	Cytoplasm	Other	0
238	Q8RG02	Cytoplasm	Other	3
239	Q8RHW9	Cytoplasm	Other	0
240	Q8RFQ1	Cytoplasm	Other	0
241	Q8RHE1	Cytoplasm	Other	1
242	Q8RF54	Cytoplasm	Other	2
243	Q8RGZ1	Cytoplasm	Other	0
244	Q8RDZ6	Cytoplasm	Other	3
245	Q8RHB0	Cytoplasm	Other	0
246	Q8RHP6	Cytoplasm	Other	0
247	Q8RH10	Cytoplasm	Other	0

248	Q8RID5	Cytoplasm	Other	0
249	Q8RGA5	Cytoplasm	Lipoprotein signal peptide (Sec/SPII) 0.9992	0
250	Q8RIP3	Cytoplasm	Other	0
251	Q8RGV4	Outer membrane	Other	0
252	Q8RGK6	Cytoplasm	Signal peptide (Sec/SPI) 0.9648	0
253	Q8RH61	Cytoplasm	Other	0
254	Q8RI31	Inner membrane	Other	11
255	Q8RII9	Cytoplasm	Other	0
256	Q8RI97	Cytoplasm	Other	0
257	Q8RIE1	Inner membrane	Other	5
258	Q8RF09	Cytoplasm	Other	0
259	Q8RHP7	Cytoplasm	Other	0
260	Q8RE00	Cytoplasm	Other	1
261	Q8RGS2	Cytoplasm	Other	0
262	Q8REL7	Inner membrane	Other	3
263	Q8RI92	Periplasm	Signal peptide (Sec/SPI) 0.9767	0
264	Q8REX6	Cytoplasm	Other	0
265	Q8RHH4	Cytoplasm	Other	0
266	Q8RFC5	Cytoplasm	Other	0
267	Q8RGW0	Cytoplasm	Other	1
268	Q8RE40	Cytoplasm	Other	1
269	Q8RIC0	Cytoplasm	Other	0
270	Q8RFS7	Outer membrane	Other	0
271	Q8REL5	Cytoplasm	Other	0
272	Q8RFJ9	Cytoplasm	Other	0

273	Q8REZ1	Cytoplasm	Other	0
274	Q8REL8	Cytoplasm	Other	0
275	Q8RGC3	Cytoplasm	Other	0
276	Q8RES6	Cytoplasm	Other	1
277	Q8RGV8	Cytoplasm	Other	0
278	Q8RF79	Cytoplasm	Other	0
279	Q8REE9	Cytoplasm	Lipoprotein signal peptide (Sec/SPII) 0.6797	1
280	Q8RFG7	Outer membrane	Signal peptide (Sec/SPI) 0.8476	0
281	Q8RF46	Outer membrane	Other	0
282	Q8RFV6	Cytoplasm	Other	0
283	Q8RFI6	Cytoplasm	Other	0
284	Q8RG54	Cytoplasm	Other	0
285	Q8RFL2	Cytoplasm	Other	0
286	Q8RE95	Cytoplasm	Other	0
287	Q8RIQ4	Cytoplasm	Other	2
288	Q8RIL7	Cytoplasm	Other	0
289	Q8RFW7	Cytoplasm	Other	0
290	Q8RFG4	Cytoplasm	Other	0
291	Q8RHV3	Cytoplasm	Other	0
292	Q8REB2	Cytoplasm	Other	0
293	Q8RH59	Cytoplasm	Other	1
294	Q8REP2	Inner membrane	Other	2
295	Q8RFJ2	Cytoplasm	Other	2
296	Q8REB3	Cytoplasm	Other	0
297	Q8RI49	Periplasm	Signal peptide (Sec/SPI) 0.9436	1

298	Q8RI95	Cytoplasm	Lipoprotein signal peptide (Sec/SPII) 0.0009	0
299	Q8RER1	Cytoplasm	Other	0
300	Q8RGC4	Cytoplasm	Other	1
301	Q8RF59	Cytoplasm	Lipoprotein signal peptide (Sec/SPII) 0.9987	1
302	Q8REJ6	Cytoplasm	Other	0
303	Q8REX8	Cytoplasm	Other	0
304	Q8RGB9	Outer membrane	Other	0
305	Q8RE01	Cytoplasm	Other	0
306	Q8RIC3	Cytoplasm	Other	0
307	Q8RF19	Periplasm	Lipoprotein signal peptide (Sec/SPII) 0.6914	0
308	Q8RI48	Cytoplasm	Signal peptide (Sec/SPI) 0.9018	1
309	Q8RHE0	Cytoplasm	Other	0
310	Q8RF83	Cytoplasm	Other	1
311	Q8RE50	Cytoplasm	Other	0
312	Q8RGV9	Cytoplasm	Other	0
313	Q8RDP4	Cytoplasm	Other	0
314	Q8RHL3	Cytoplasm	Other	1
315	Q8RH94	Cytoplasm	Other	0
316	Q8RGW2	Cytoplasm	Other	0
317	Q8RFW6	Cytoplasm	Other	3
318	Q8RFK7	Cytoplasm	Other	0
319	Q8RDN2	Cytoplasm	Other	0
320	Q8RHW2	Cytoplasm	Other	0

321	Q8RE27	Cytoplasm	Other	0
322	Q8RIK7	Extracellular	Other	0
323	Q8RFM6	Cytoplasm	Other	0
324	Q8RG60	Inner membrane	Other	4
325	Q8RE17	Cytoplasm	Other	0
326	Q8RFH0	Cytoplasm	Other	0
327	Q8REM3	Inner membrane	Other	6
328	Q8RIB4	Cytoplasm	Other	0
329	Q8RFE3	Cytoplasm	Other	0
330	Q8RDX6	Cytoplasm	Other	0
331	Q8REK3	Cytoplasm	Other	2
332	Q8RGY3	Cytoplasm	Other	0
333	Q8RH29	Cytoplasm	Other	0
334	Q8RG37	Cytoplasm	Signal peptide (Sec/SPI) 0.9411	0
335	Q8RH93	Cytoplasm	Other	0
336	Q8REV5	Cytoplasm	Other	0
337	Q8RFB9	Cytoplasm	Other	0
338	Q8REP9	Cytoplasm	Other	0
339	Q8RGQ6	Periplasm	Signal peptide (Sec/SPI) 0.7967	1
340	Q8REQ5	Cytoplasm	Other	0
341	Q8RHN1	Cytoplasm	Other	0
342	Q8RHE8	Cytoplasm	Other	1
343	Q8REI4	Cytoplasm	Other	0
344	Q8REN7	Inner membrane	Other	5
345	Q8RGA1	Periplasm	Lipoprotein signal peptide (Sec/SPII) 0.9985	0

346	Q8RG08	Inner membrane	Other	4
347	Q8RFA5	Cytoplasm	Other	0
348	Q8RG74	Inner membrane	Other	1
349	Q8REC6	Cytoplasm	Other	0
350	Q8REX1	Cytoplasm	Other	0
351	Q8RGC5	Cytoplasm	Other	3
352	Q8RI91	Cytoplasm	Other	1
353	Q8RF75	Cytoplasm	Other	2
354	Q8RH91	Cytoplasm	Other	0
355	Q8RGY1	Cytoplasm	Other	2
356	Q8RGH9	Cytoplasm	Other	0
357	Q8RHH0	Cytoplasm	Other	0
358	Q8REW6	Cytoplasm	Other	0
359	Q8RH76	Extracellular	Signal peptide (Sec/SPI) 0.9503	0
360	Q8RH01	Cytoplasm	Other	2
361	Q8RHR5	Inner membrane	Other	7
362	Q8RH12	Inner membrane	Other	3
363	Q8RGE8	Cytoplasm	Other	1
364	Q8RE96	Cytoplasm	Other	2
365	Q8RGY4	Cytoplasm	Other	0
366	Q8RFW4	Inner membrane	Other	1
367	Q8RIH9	Cytoplasm	Other	1
368	Q8RI40	Cytoplasm	Other	0
369	Q8RFK8	Cytoplasm	Other	0
370	Q8RHC5	Cytoplasm	Other	0
371	Q8RIC7	Cytoplasm	Other	0

372	Q8RIM4	Inner membrane	Other	3
373	Q8RG38	Outer membrane	Other	1
374	Q8RH73	Cytoplasm	Other	2
375	Q8RF03	Inner membrane	Other	4
376	Q8RHP8	Inner membrane	Other	2
377	Q8RGG0	Cytoplasm	Other	2
378	Q8RHC3	Cytoplasm	Lipoprotein signal peptide (Sec/SPII) 0.9732	0
379	Q8RFH7	Outer membrane	Signal peptide (Sec/SPI) 0.896	1
380	Q8RHV9	Cytoplasm	Other	0
381	Q8RH69	Cytoplasm	Other	0
382	Q8RGP0	Cytoplasm	Other	1
383	Q8RGP7	Cytoplasm	Other	2
384	Q8RDV5	Cytoplasm	Other	0
385	Q8RE05	Cytoplasm	Other	1
386	Q8RGX9	Inner membrane	Other	4
387	Q8RGQ2	Cytoplasm	Other	0
388	Q8RF70	Cytoplasm	Other	0
389	Q8RI41	Cytoplasm	Other	0
390	Q8RDW0	Cytoplasm	Other	0
391	Q8RF29	Cytoplasm	Other	0
392	Q8RIA8	Cytoplasm	Other	0
393	Q8R6H9	Cytoplasm	Lipoprotein signal peptide (Sec/SPII) 0.999	0
394	Q8R6K0	Cytoplasm	Other	0
395	Q8R6I0	Cytoplasm	Other	0

396	Q8R6K1	Cytoplasm	Other	1
397	Q8RHM0	Periplasm	Other	0
398	Q8RHF0	Cytoplasm	Other	0
