# Supplementary material for: Functional annotation of uncharacterized proteins from Fusobacterium nucleatum: identification of virulence factors
Source: Genomics Inform. 2023 Jun 30;21(2):e21. doi: 10.5808/gi.22065 (PMC10326533; doi:10.5808/gi.22065)
Supplement: Supplementary Table 5. — Functional domains identified in uncharacterized proteins of Fusobacterium nucleatum [file gi-22065-Supplementary-Table-5.pdf]

Supplementary Table 5. Functional domains identified in uncharacterized proteins of *Fusobacterium nucleatum*

S. No.	Accession ID	InterProScan	Motif	SMART	HMMER	CDART	Probable function
1	Q8R6J3	-	Anti-repressor SinI, MerR, DNA binding, DUF6115, DUF6312	-	-	-	-
2	Q8R669	Nucleoside phosphorylase superfamily	Radical SAM superfamily, Tetrahydrofolate dehydrogenase/cyclohydrolase, NAD(P)-binding domain	Elp3 (elongator protein 3, MiaB family, Radical SAM)	-	Nucleoside phosphory lase-I superfamil y, Photo_TT _lyase	Enzyme
3	Q8RDL8	-	-	No result because of too short sequence	-	-	-
4	Q8RDM0	Lipoprotein localization LolA/LolB/Lpp X	-	-	-	LolA_fold-like	-
5	Q8RDN3	-	-	-	-	-	-
6	Q8RDP1	Kelch-type beta propeller, Cyclically-permuted mutarotase family	Kelch motif, Galactose oxidase, central domain, Domain of unknown function (DUF5074)	Kelch _4q3	Galactose oxidase, central domain (Kelch 4)	Cyclically-permuted mutarotas e family protein	Binding protein
7	Q8RDR8	-	-	-	-	-	-
8	Q8RDV4	-	-	-	-	-	-

9	Q8RDW3	Autotransporter beta-domain superfamily	Uncharacterized protein conserved in bacteria (DUF2345)	-	-	Outer membrane autotransporter barrel domain superfamily	-
10	Q8RDY5	SseB protein N-terminal domain (SseB_N), SseB protein, C-terminal (SseB_C)	SseB Protein C-terminal domain, SseB protein N-terminal domain, Caspase recruitment domain	SseB_C, SseB	SseB protein N-terminal domain (SseB), SseB protein C-terminal domain (SseB_C)	SseB superfamily, SseB_C superfamily	Regulatory protein (enhance serine sensitivity)
11	Q8RE02	-	STAS-like domain of unknown function (DUF4325), Beclin-1 BH3 domain, Bcl-2 interacting, Protein RETICULATA-related	-	-	-	-
12	Q8RE16	-	TPM_Phosphatase domain, Chordopoxvirus A13L protein, SOCE-associated regulatory factor of calcium homoestasis, ABC-2_membrane_3	-	-	-	-
13	Q8RE33	-	-	-	-	-	-
14	Q8RE35	DKNYY family	DKNYY family, Phage integrase, N-terminal SAM-like domain, Apolipoprotein O	DKNYY	DKNYY family	DKNYY	Function unknown
15	Q8RE36	DKNYY	DKNYY family, GRAM domain	DKNYY	DKNYY family	DKNYY	Function unknown
16	Q8RE37	DKNYY family	DKNYY family, Zinc-finger like, probable DNA-binding	-	DKNYY family	DKNYY family	Function unknown
17	Q8RE38	DKNYY family	DKNYY	DKNYY	DKNYY family	DKNYY	Function unknown

18	Q8RE39	DKNYY family	DKNYY family, Seryl-tRNA synthetase N-terminal domain, Viral late protein H2, M penetrans paralogue family 26	DKNYY	DKNYY family	DKNYY, PLN02678 (seryl-tRNA synthetase)	Function unknown
19	Q8RE61	-	-	-	-	-	-
20	Q8RE69	-	Glycine zipper, YMGG-like Gly-zipper, Glycine-zipper domain, Glycine zipper 2TM domain, Enterobacterial TraT complement resistance protein	Gly-zipper_OMP	Glycine-zipper_OMP	-	Membrane protein
21	Q8RE90	-	-	-	-	-	-
22	Q8RE93	YwqG-like superfamily, Protein of unknown function DUF1963	Domain of unknown function (DUF1963), Family of unknown function (DUF5891)	DUF1963	Domain of unknown function DUF1963	DUF1963	Function unknown
23	Q8REA1	-	-	-	-	-	-
24	Q8REA8	-	Pex24p integral peroxisomal membrane peroxin, FliP family, DUF4191	-	-	-	-
25	Q8REA9	Autotransporter beta-domain superfamily	Outer membrane protein beta-barrel domain, Autotransporter beta-domain, Attacin, C-terminal region, *Legionella pneumophila* major outer membrane protein precursor	-	-	-	-
26	Q8REB4	-	Tctex-1 family, Methanogen output domain 1	-	-	-	-
27	Q8REB5	-	Protein of unknown function (DUF3217)	-	-	-	-
28	Q8REB6	-	-	-	-	-	-

29	Q8REB7	-	SKG6 (transmembrane alpha-helix domain), Reticulon, Cbl_N3 (CBL proto-oncogene N-terminus, SH2- like domain), TMP_2 prophage tail length tape measure protein	-	-	-	-
30	Q8REB8	-	Polysulphide reductase, NrfD	-	-	-	-
31	Q8REC0	-	Heavy metal associated domain 2, Protein of unknown function (DUF2498), Thermopsin	-	Heavy metal associated domain 2 (HMA_2)	-	-
32	Q8REC7	CRISPR-associated protein Cas7/Cst2/DevR CRISPR-associated protein Cas7, subtype I-B/Tneap	DevR, CRISPR-associated negative auto-regulator DevR/Csa2	DevR	CRISPR-associated negative auto-regulator DevR/Csa2 (DevR)	Cas7_I superfamily	Defense, binding protein
33	Q8REC8	-	-	-	-	Cas5_I superfamily	-
34	Q8RED4	-	Phage-Barnase-EndoU-ColicinE5/D-RelE like nuclease4	-	-	-	-
35	Q8RED5	-	Mitochondrial ribosomal subunit, Type III Restriction/modification enzyme methylation subunit	-	-	-	-
36	Q8REF3	-	-	-	-	-	-
37	Q8REG3	FldB/FldC dehydratase alpha/beta subunit, Unintegrated	2-hydroxyglutaryl-CoA dehydratase, D-component, CoA enzyme activase uncharacterized domain (DUF2229)	HGD-D	2-Hydroxyglutaryl-CoA dehydrated, D-component (HGD-D)	HGD-D superfamily	HGD-D enzyme

38	Q8REH2	-	Protein of unknown function (DUF1700)	-	-	-	-
39	Q8REI8	DUF1007	DUF1007	DUF1007	Protein of unknown functionDUF1007	DUF1007	Function unknown
40	Q8REK1	-	Family of unknown function (DUF6290), Cysteine rich domain with multi zinc binding regions	-	Family of unknown function (DUF6290)	-	-
41	Q8REK2	-	Rabenosyn Rab binding domain, N-terminal domain of reverse transcriptase, RFX5 N-terminal domain	-	-	-	-
42	Q8REK5	-	Transmembrane protein 51, LPXTG cell wall anchor motif, Conotoxin, African swine fever virus J13L protein, Family of unknown function (DUF5775), Putatibve PepSY_TM-like	-	-	-	-
43	Q8REK8	DUF1385	Protein of unknown function (DUF1385), Domain of unknown function (DUF6391)	DUF1385	Protein of unknown function DUF1385	DUF1385	Function unknown
44	Q8REL2	-	Zinc finger, C2H2 type, RloB-like protein	-	-	-	-
45	Q8REM4	Alpha/Beta hydrolase fold, GPI inositol-deacylase PGAP1-like	PGAP1-like protein, Putative serine esterase (DUF676), Alpha/beta hydrolase family	PGAP1	PGAP1-like protein	Abhydrolase superfamily	Enzyme
46	Q8REM6	-	-	-	-	-	-
47	Q8REN4	-	Lysine exporter LysO, 2-hydroxycarboxylate transporter family	-	-	-	-
48	Q8REN5	Protein of unknown function DUF3100	Protein of unknown function (DUF3100)	DUF3100	Protein of unknown function DUF3100	DUF3100	Function unknown

49	Q8REP0	-	BMP and activin membrane-bound inhibitor homolog C-terminus, Preprotein translocase subunit	-	-	-	-
50	Q8REP4	-	Protein of unknown function (DUF3139)	-	-	-	-
51	Q8REP7	-	OST3/OST6 family, transporter family, Positive regulator of sigma(E), RseC/MucC, UbiA prenyltransferase family, Yip1 domain	-	-	-	-
52	Q8REQ4	Bacteriophage phiKZ, Orf197	Protein of unknown function (DUF3307), ATP synthase I chain	DUF3307	Protein of unknown function DUF3307	DUF3307	Function unknown
53	Q8RER4	Colicin-V Production, CvpA	Colicin V production	Colicin _V	Colicin Vproductio n protein (Colicin_V)	Colicin_V	Biosynthesis
54	Q8RES9	-	-	-	-	-	-
55	Q8RET2	-	-	-	-	-	-
56	Q8RET3	DUF3592	Protein of unknown function (DUF3592)	DUF3592	Protein of unknown function DUF3592	DUF3592	Function unknown
57	Q8REU4	-	Predicted membrane protein (DUF2208), Poxvirus entry protein complex L1 and I2, MFS/Sugar transport protein, Protein of unknown function (DUF3099), Domain of unknown function (DUF4190), Domain of unknown function (DUF4191), Domain of unknown function (DUF6377) WW domain-binding protein 1, Protein of unknown function (DUF3169)	-	-	-	-

58	Q8REW7	-	-	-	-	Vacuolating cyotoxin (VacA) superfamily	-
59	Q8REX3	-	-	-	-	-	-
60	Q8REX4	-	GHKL domain, Nucleoporin protein Ndc1-Nup, Peptidase M66	-	-	-	-
61	Q8REY7	DUF4198, Carboxypeptidase-like, regulatory domain superfamily	Domain of unknown function (DUF4198), Dioxygenase, Carboxypeptidase regulatory-like domain	DUF4198	Domain of unknown function DUF4198	DUF4198	Function unknown
62	Q8REZ3	-	Succinylglutamate desuccinylase/ Aspartoacylase family	-	-	Peptidase M14 like superfamily	-
63	Q8REZ5	Poly-gamma-glutamate system protein	Poxvirus entry protein complex L1 and I2, Family of unknown function (DUF5577)	-	-	Poly-gamma-glutamate system superfamily	-
64	Q8REZ8	-	MASE 3 membrane-associated sensor domain, DUF2619, DUF308	-	-	DNAQ-like-3’-5’ exonuclease domain superfamily	-
65	Q8RF05	-	D-ala D-ala ligase C-terminus, Protein of unknown function DUF3658	-	-	-	-

66	Q8RF08	-	Family of unknown function (DUF6057), Protein of unknown function (DUF3500), Kinetochore component, CENP-R, Inclusion membrane protein D, Family of unknown function (DUF6418)	-	-	-	-
67	Q8RF26	-	Flavodoxin_1	-	-	-	-
68	Q8RF27	-	Family of unknown function (DUF5928)	-	-	-	-
69	Q8RF36	-	-	-	-	-	-
70	Q8RF53	-	-	-	-	-	-
71	Q8RF56	-	ABC-2 family transporter protein	-	-	-	-
72	Q8RF58	-	-	-	-	-	-
73	Q8RF72	-	-	-	-	-	-
74	Q8RF74	-	Protein of unknown function (DUF1493)	-	-	-	-
75	Q8RF78	-	-	-	-	-	-
76	Q8RF82	-	-	-	-	-	-
77	Q8RF86	ATP-dependent DNA helicase RecG, C-terminal domain, Schlafen, AlbA_2 domain superfamily, Winged Helix DNA- binding domain superfamily	Putative DNA-binding domain, Putative ATP-dependent DNA helicase RecG C-terminal, Ribonuclease R winged-helix domain, Winged helix-turn-helix DNA-binding, Family of unknown function (DUF5815), HTH domain Helix-turn-helix domain, FaeA-like protein	AlbA_2, HATPase_c_4	Putative DNA-binding domain (AlbA_2), Putative ATP-depndent DNA helicase RecG C-terminal (HATPase_c_4)	COG2865 superfamily	Binding protein
78	Q8RF87	-	Protein of unknown function (DUF3139)	-	-	-	-
79	Q8RFB6	-	-	-	-	-	-

80	Q8RFD4	RelB antitoxin/Antitoxin DinJ, Arc-type ribbon-helix-helix	RelB antitoxin, DUF6364	RelB	RelB antitoxin	RelB	Regulatory protein
81	Q8RFE5	-	Selenoprotein S (SelS), Uncharacterised protein family UPF0542	-	-	TolA superfamily	-
82	Q8RFF3	Putative L,D-transpeptidase tautomerase, YkuD_2	L,D-transpeptidase catalytic domain	YkuD _2	L, D-transpeptidase catalytic domain (YkuD_2)	YkuD_like superfamily	Enzyme
83	Q8RFF4	DUF1576	DUF1576, SecD_SecF (Protein export membrane protein), StbA protein	DUF1576	Protein of unknown function DUF1576	DUF1576	Function unknown
84	Q8RFF9	Domain of unknown function, DUF1456	Protein of unknown function (DUF1456)	DUF1456	Protein of unknown function DUF1456	Uncharacterized conserved protein YehS, DUF 1456 family	Function unknown
85	Q8RFH1	-	-	-	-	-	-
86	Q8RFH2	Winged Helix DNA-binding domain superfamily	Fic/DOC family N-terminal, Helix-turn-helix domain, HTH domain, MarR family, Winged helix-turn-helix DNA-binding, Crp-like Helix-turn-helix domain, Sugar-specific transcriptional regulator TrmB, MarR family	-	-	Fic_N Superfamily	-
87	Q8RFH5	-	Domain of unknown function (DUF4134), Importin 13 repeat	-	-	-	-
88	Q8RFH9	-	Mucin-like glycoprotein, O-antigen ligase like membrane protein, FtsH Extracellular	-	-	-	-

89	Q8RFI1	MORN motif	‘Cold-shock’ DNA -binding domain	MORN	MORN repeat	YwqK Superfamily	Function unknown
90	Q8RFN1	DsrEFH-like	DsrE/DsrF-like family, Peroxisomal biogenesis protein family, Family of unknown function (DUF6394)	-	-	-	-
91	Q8RFN8	-	-	-	-	Tudor domain superfamily	-
92	Q8RFQ0	MSMEG_1276-like_Nucleoside triphosphate pyrophosphohydrolase domain	Protein of unknown function (DUF4127), Domain of unknown function (DUF6449), YpjP-like protein, Est1 DNA/RNA binding domain	-	-	Nucleoside triphosphate pyrophosphohydrolase MazG-like domain superfamily	-
93	Q8RFQ3	Campylobacter Phage CGC-2007, Cje0229	Protein of unknown function (DUF1353), Phospholipase A2-like domain, Uncharacterized conserved protein (DUF2358)	Protein of unknown Function (DUF1353)	Protein of unknown function DUF1353	DUF1353	Function unknown
94	Q8RFR7	-	-	-	-	-	-
95	Q8RFS5	-	Domain of unknown function (DUF4367)	-	-	-	-
96	Q8RFT5	-	DNA polymerase II intein Domain IV	-	-	-	-
97	Q8RFT7	-	Lipoprotein leucine-zipper	-	-	-	-
98	Q8RFU1	LpxI, C-terminal LpxI N-terminal domain LpxI, C-terminal domain superfamily	LpxI C-terminal domain, LpxI N-terminal domain	DUF1009	LpxI_N-terminal domain (LpxI_N), LpxI_C-terminal domain (LpxI_C)	DUF1009	Enzyme

99	Q8RFU6	-	-	-	-	-	-
100	Q8RFX3	-	-	-	-	-	-
101	Q8RFZ2	-	M penetrans paralogue family 26, G protein-coupled glucose receptor regulating Gpa2 C-term, PgaD-like protein	-	-	-	-
102	Q8RFZ5	-	-	-	-	-	-
103	Q8RG13	-	-	-	-	-	-
104	Q8RG23	Antitoxin ParD superfamily, Ribbon-helix-helix	Family of unknown function (DUF6290), Antitoxin ParD, Domain of unknown function (DUF4460), Protein of unknown function (DUF1778), Ribbon-helix-helix protein, copG family, Chromatin modification-related protein EAF7, Phage minor capsid protein 2, DsrC like protein, Cysteine rich domain with multizinc binding regions, Tc3 transposase	-	Family of unknown function (DUF6290)	Ribbon-helix-helix domains of transcription repressor CopG, Nickel responsive transcription factor NikR- like superfamily	Repressor Regulatory protein
105	Q8RG27	-	-	-	-	-	-
106	Q8RG66	-	C-terminal domain of RACo the ASKHA domain	-	-	-	-
107	Q8RG68	-	-	-	-	-	-
108	Q8RG71	-	Protein of unknown function (DUIF815), Tsi6	-	-	HsdS superfamily	-
109	Q8RG81	-	-	-	-	-	-
110	Q8RG93	Protein of unknown function DUF4391	Domain of unknown function (DUF4391)	DUF4391	Domain of unknown function DUF4391	DUF4391	Function unknown
111	Q8RG95	-	-	-	-	-	-

112	Q8RGC0	Putative exopolysaccharide exporter PelG	Putative exopolysaccharide Exporter (EPS-E)	PelG	Putative exopolysaccharide Exporter (EPS-E) PelG	PelG superfamily Putative exoploysaccharide exporter (EPS-E)	Transport protein
113	Q8RGC2	-	Fungal N-terminal domain of STAND proteins	-	-	-	-
114	Q8RGE9	-	-	-	-	-	-
115	Q8RGF5	-	-	-	-	PRK12704 superfamily	-
116	Q8RGF9	Protein of unknown function DUF1877, YfbM-like superfamily	Domain of unknown function DUF1877	DUF1877	Domain of unknown function DUF1877	DUF1877	Function unknown
117	Q8RGG1	-	-	-	-	-	-
118	Q8RGK4	-	-	-	-	-	-
119	Q8RGL1	AB_hydrolase MlaA lipoprotein	Abhydrolase_1, Abhydrolase_3 (Alpha/Beta Hydrolase fold), Lipase_3	-	-	-	-

120	Q8RGM7	Adhesion protein FadA	Adhesion protein FadA, Poly (R)-Hydroxyalkanoic acid synthase subunit, Spindle and kinetochore-associated protein 2, Putative neutral zinc metallopeptidase, Hepatic lectin, N-terminal domain, TMPIT-like protein, Nitrate and nitrite sensing, Carboxypeptidase Y pro-peptide, JNK_SAPK-associated protein-1, Finbrinogen alpha/beta chain family, Flagellar assembly protein A, Exonuclease VII, large subunit, Protein of unknown function, DUF412	FadA	Adhesion Protein FadA	FadA Superfamily	Others
121	Q8RGP8	Predicted amidinotransferase, FN0238 type	N,N dimethylarginine dimethylhydrolase, eukaryotic (DDAH_eukar)	Amidinotransf	N,N, dimethylarginine dimethylhydrolase, eukaryotic (DDAH_eukar)	Amidinotransferase superfamily	Enzyme
122	Q8RGQ3	-	Cytochrome C and quinol oxidase polypeptide I, 5TM C-terminal transporter carbon starvation CstA, Family of unknown function (DUF5305)	-	-	Major facilitator superfamily (MFS)	-
123	Q8RGQ7	-	-	-	-	-	-

124	Q8RGQ9	Nucleotide binding domain (NBD_C), DUF1537	Sugar-binding N-terminal domain (SBD_N), Nucleotide-binding C-terminal domain (NBD_C)	DUF1537, DUF1357_C	Sugar-binding N-terminal domain (SBD_N), Nucleotide-binding C-terminal domain (NBD_C)	Uncharacterized conserved protein, YgbK DUF 1537 family	Binding protein
125	Q8RGU2	DUF2813 OLD protein-like, TOPRIM domain	Protein of unknown function (DUF2813)	-	Protein of unknown function DUF2813	YbjD superfamily	Function unknown
126	Q8RGW1	-	7TMR-DISM_7TM (7TM diverse intracellular signaling)	-	-	-	-
127	Q8RGY0	-	-	-	-	-	-
128	Q8RGY2	-	-	-	-	-	-
129	Q8RGZ2	-	-	-	-	-	-
130	Q8RGZ9	-	-	-	-	-	-
131	Q8RH06	-	-	-	-	-	-
132	Q8RH09	-	-	-	-	-	-
133	Q8RH22	-	-	-	-	-	-

134	Q8RH50	-	Transcriptional activator, Baculovirus P24 capsid protein, Apolipoprotein A1/A4/E domain, Poly(hydroxyalcanoate) granule associated protein, Baculovirus polyhedron nenvelope protein, PEP,C terminus, Golgin subfamily A member 5, Protein of unknown function (DUF1664), Prominin, Apolipophorin-III precursor (apoLp-III), Cobalamin adenosyltransferase, Apoptosis antagonizing transcription factor, COG complex component, COG2, Centromere protein H (CENP-H), Tweety, Prefoldin subunit	-	-	-	-
135	Q8RH72	-	Protein of unknown function (DUF3232), Family of unknown function (DUF5926), gp58-like protein, D-xylulose 5-phosphate/D-fructose 6-phosphate phosphoketolase, Stage III sporulation protein AF (spore_III_AF)	-	-	-	-
136	Q8RH77	DUF4132 DUF5724	DUF5724, DUF4132	DUF4132	Family of unknown function DUF5724, Domain of unknown function DUF4132	CAS12d, DUF5724, DUF4132	Function unknown

137	Q8RH78	Peptidase_C45, Nucleophile aminohydrolases, N-terminal	Acyl-coenzyme A:6-aminopenicillanic acid acyl-transferase (AAT), Linear amide C-N hydrolases, chologlycine hydrolase family (CBAH), XPG domain containing (XPG_I_)	AAT	Acyl-coenzyme A: 6-aminopencillanic acid acyl-transferas (AAT)	COG4927 superfamily	Enzyme
138	Q8RH83	-	-	-	-	-	-
139	Q8RHC2	-	YonK Protein	-	-	-	-
140	Q8RHD8	Protein of unknown function, DUF454	Protein of unknown function (DUF454), Heterokaryon incompatibility protein Het-C, Uncharacterized conserved protein (DUF2304)	DUF454	Protein of unknown function DUF454	DUF454	Function unknown
141	Q8RHE6	-	Histone chaperone Rttp106-like, Phosphoinositide phosphatase insertion domain, chi-Conotoxin or t superfamily	-	-	-	-
142	Q8RHE9	Type IV pilus inner membrane component PilN	PilN, DUF4446, Apg6_N, DivIC, GrpE, DUF2681, Knl1_RWD_C, Sec 20, SUR7, TolA_bind_tri, DUF948, Atg11_middle, DuF1664, DUF3450, DUF4140, Ax_dynein_light, Nucleoside_tran, Syntaxin-6_N	PilN	Fimbrial assembly protein PilN	DUF460, PilN	Pili assembly protein
143	Q8RHG1	-	-	-	-	-	-
144	Q8RHG5	-	Coronavirus M matrix/glycoProtein, Fusaric acid resistance protein family, Keratinocyte-associated gene product	-	-	-	-
145	Q8RHP4	-	Domain of unknown function (DUF3512), Staphylococcal nuclease homologue (SNase)	-	-	-	-

146	Q8RHQ2	Macro Domain	Macro domain	Appr-1”-p processing enzyme	Macro domain	Macro domain superfamily	Regulatory protein
147	Q8RHR0	-	-	-	-	-	-
148	Q8RHR2	-	EcoEI R protein C-terminal, Formylmethanofuran-tetrahydromethanopterin formyltransferase	-	-	-	-
149	Q8RHR3	Armadillo-type fold, Armadillo-like helical	HEAT repeats, Domain of unknown function (DUF4807)	HEAT_2	HEAT repeats (HEAT_2)	HEAT_2 superfamily	Transport protein
150	Q8RHR6	-	Haemolysin XhlA	-	-	-	-
151	Q8RHS6	Restriction endonuclease, type II, AlwI	AlwI restriction endonuclease, Intein splicing domain, 5-formyltetrahydrofolate cyclo-ligase family	-	AlwI restriction endonuclease	AlwI restriction endonuclease	Enzyme
152	Q8RHS9	-	-	-	-	-	-
153	Q8RHU8	-	-	-	-	-	-
154	Q8RHW0	-	-	-	-	-	-
155	Q8RHW8	-	FAM76 protein, Ubiquinol-cytochrome-c reductase complex assembly factor 3, Noc2p family	-	-	-	-
156	Q8RHX5	-	Putative GlcNAc-1 phosphotransferase regulatory domain, 50S ribosome-binding GTPase, Molybdopterin guanine dinucleotide synthesis protein B	-	-	-	-
157	Q8RHY8	-	-	-	-	-	-
158	Q8RHZ3	-	AAA domain	-	-	-	-
159	Q8RI03	-	Protein of unknown function (DUF3828), Collagen binding domain, Fungal domain of unknown function (DUF1750), DUSP domain	-	-	-	-

160	Q8RI09	-	-	-	-	-	-
161	Q8RI11	-	-	-	-	-	-
162	Q8RI28	-	Protein of unknown function (DUF2606)	-	-	-	-
163	Q8RI34	-	-	-	-	-	-
164	Q8RI90	Apc36109-like domain superfamily, Protein of unknown function (DUF1871)	Domain of unknown function (DUF1871), HEPN domain, Protein of unknown function (DUF507)	DUF1871	Domain of unknown function DUF1871	-	Function unknown
165	Q8RI98	-	Domain of unknown function (DUF6377), Up-regulated during septation, Lipopolysaccharide assembly protein A domain	-	-	-	-
166	Q8RIA4	-	-	-	-	-	-
167	Q8RIB1	-	-	-	-	GumC superfamil y	-
168	Q8RIB2	-	LicD Family	-	-	-	-
169	Q8RIC8	-	-	-	-	-	-
170	Q8RID7	Toxin-antitoxin system, RelE/ParE toxin domain superfamily	RelE-like toxin of type II toxin-antitoxin system HigB	-	-	-	-

171	Q8RID9	ATP-dependent DNA helicase RecG, C-terminal domain Schlafen, AlbA_2 domain, Schlafen, AlbA_2 domain superfamily RecG, C-terminal domain superfamily	Putative ATP-dependent DNA helicase RecG C-terminal, Putative DNA-binding domain	AlbA_2, HATPas_c_4	Putative DNA binding domain (AlbA_2), Putative ATP-dependent DNA helicase RecG C-terminal (HATPase_c_4)	COG2865 superfamily	Binding and regulatory protein
172	Q8RII7	Metallophosphoesterase, YmdB-like	YmdB-like protein, Calcineurin-like phosphoesterase	YmdB	YmdB-like protein	Metallophosphatase superfamily	Enzyme
173	Q8RIJ3	Uncharcterised conserved protein UCP015278	Uncharacterized protein conserved in bacteria (DUF2247), PAS fold	DUF2247	Uncharacterized protein conserved in bacteriaDUF2247	DUF2247	Function unknown
174	Q8RIJ4	-	-	-	-	-	-
175	Q8RIK2	-	Renin receptor-like protein, Fusaric acid resistance protein family	-	-	-	-
176	Q8RIK4	Protein of unknown function DUF819	Protein of unknown function (DUF819), Protein of unknown function (DUF485)	-	Protein of unknown function DUF819	DUF819	Function unknown
177	Q8RE79	-	-	-	-	-	-
178	Q8RE80	O-antigen_ligase-related	O-antigen ligase	Wzy_C	O-antigen ligase (Wzy_C)	Wzy_C superfamily	Enzyme
179	Q8REC1	-	Heavy metal associated domain 2, Carboxyltransferase domain, sub domain C and D ATP synthase E chain	-	Heavy Metal associated Domain-2 (HMA_2)	-	-

180	Q8RED3	-	Poxvirus T4 Protein, N terminus	-	-	-	-
181	Q8REK4	-	Nucleopolyhedrovirus P10 protein, Actin cytoskeleton-regulatory complex protein END3, Protein of unknown function (DUF4446)	-	-	-	-
182	Q8REK7	Transcription regulator Rrf2 Winged Helix-like DNA-binding domain superfamily, Winged helix DNA-binding domain superfamily	Iron-dependent transcriptional regulator, LlaMI restriction endonuclease, Sugar-specific transcriptional regulator TrmB, Linker histone H1 and H5 family, MarR Family	Rrf2	Iron dependent transcriptional regulator (Rrf2)	HTH superfamily	Regulatory proteins
183	Q8REQ2	-	-	-	-	-	-
184	Q8REQ3	SatD family	SatD family (SatD), Minimal CRISPR polymerase domain	SatD	SatD family	SatD superfamily	Acid resistance (Other)
185	Q8RET5	-	Protein of unknown function (DUF1450), Domain of unknown function (DUF5590)	-	-	-	-
186	Q8RF55	-	-	-	-	-	-
187	Q8RF57	-	-	-	-	-	-
188	Q8RF73	-	Protein of unknown function (DUF2663), Glycoprotein VP7	-	-	-	-
189	Q8RF84	-	Peroxisomal biogenesis protein family	-	-	-	-
190	Q8RF95	-	-	-	-	-	-
191	Q8RFF2	-	Sporulation protein YhaL	-	-	-	-
192	Q8RFI2	TTHA1013/TTHA0281-like	HicB_like antitoxin of bacterial toxin-antitoxin system, NleF caspase inhibitor, Alpha/Beta-hydrolase family	-	-	PTZ00419 superfamily	-
193	Q8RFK6	-	Domain of unknown function (DUF5105), Protein of un known function (DUF1366)	-	-	-	-
194	Q8RFM9	-	-	-	-	-	-

195	Q8RFS4	-	Domain of unknown function (DUF4367)	-	-	-	-
196	Q8RFV9	-	-	-	-	-	-
197	Q8RFW1	-	Protein of unknown function (DUF3723) Emp24/gp25L/P24 family/GOLD	-	-	-	-
198	Q8RG53	Tetratricopeptide-like helical domain superfamily	TPR repeat, Tetratricopeptide repeat, Cytidine and deoxycytidylate deaminase zinc-binding region	-	-	YbgF superfamliy	Binding Protein
199	Q8RGQ5	Protein of unknown function DUF4241	Protein of unknown function DUF 4241	DUF4241	Protein of unknown function DUF4241	DUF4241 Superfamily	Function unknown
200	Q8RGU3	-	TMEM154 protein family, Family of unknown function (DUF5383), Domain of unknown function (DUF3377), Orf78 (ac78)	-	-	-	-
201	Q8RGW8	Phosphoribosyltransferase-like	Uracil phosphoribosyltransferase, Phosphoribosyl transferase, Phosphoribosyl transferase domain	-	-	-	-
202	Q8RGX2	-	-	-	-	-	-
203	Q8RH27	-	Domain of unknown function (DUF4258)	-	-	-	-
204	Q8RH75	-	-	-	-	-	-
205	Q8RH79	-	-	-	-	-	-
206	Q8RHC4	-	-	-	-	-	-
207	Q8RHE7	-	Prokaryotic N-terminal methylation motif	-	-	-	-
208	Q8RHK2	-	RHS repeat, Protein of unknown function (DUF3022)	-	-	-	-
209	Q8RHL9	-	-	Elp3	-	COG1244 Superfamily	-
210	Q8RHR1	-	Protein of unknown function (DUF3592)	-	-	-	-

211	Q8RHT3	-	Domain of unknown function (DUF5004)	-	-	TamB superfamily	-
212	Q8RHV0	-	-	-	-	-	-
213	Q8RHV7	-	Domain of unknown function (DUF5077), Family of unknown function (DUF6314), Septin, Glycosyl hydrolase family 65, C-terminal domain, Carbohydrate esterase 2 N-terminal	-	-	-	-
214	Q8RHX0	MFS transporter superfamily	Major Facilitator Superfamily	-	-	-	-
215	Q8RI20	DUF4304	Domain of unknown function (DUF4304), Putative conjugal transfer nickase/helicase TraI C-term	DUF4304	Domain of unknown function DUF4304	DUF4304	Function unknown
216	Q8RI29	Protein of unknown function DUF4198	Domain of unknown function (DUF4198), Dioxygenase, Carboxypeptidase regulatory-like domain, Tombusvirus movement protein, YtkA-like	DUF4198	Domain of unknown function DUF4198	DUF4198	Unknown Function
217	Q8RI81	-	-	-	-	PhzC-PhzF superfamily	-
218	Q8RIF3	-	-	-	-	-	-
219	Q8RIP2	Adhesion protein FadA	Adhesion protein FadA, AAA domain, Zinc-uptake complex component A periplasmic	FadA	Adhesion protein FadA	FadA	Other (adhesion protein)
220	Q8RIP4	-	Domain of unknown function (DUF4969), Septum formation initiator, Protein of unknown function DUF262, Short chain dehydrogenase, Kinetochore complex Fta4 of Sim4 subunit, or CENP-50, GNT-I family, Protein of unknown function (DUF4239)	-	-	-	-

221	Q8RIR0	-	-	No result because of too short sequence	-	-	-
222	Q8RGD0	-	Protein of unknown function (DUF1189)	-	-	-	-
223	Q8RFW3	-	Archaeal Holliday junction resolvase (hjc)	-	-	-	-
224	Q8RI22	-	Family of unknown function (DUF6162)	-	-	-	-
225	Q8RGU6	Protein of unknown function DUF1667, CPE0013-like superfamily	Protein of unknown function DUF1667	DUF1667	Protein of unknown function DUF1667	COG3862 Superfamily	Function unknown
226	Q8REU5	-	Ubc7p-binding region of Cue1	-	-	-	-
227	Q8RF11	-	Dihydrodipicolinate synthetase family, Cyclin-dependent kinase inhibitor 3 (CDKN3)	-	-	HolA superfamily	-
228	Q8RF13	Putative L,D-transpeptidase tautomerase (YkuD_2)	L,D-transpeptidase catalytic domain	YkuD_2	L,D-transpeptidase catalytic domain (YkuD_2)	YkuD_like superfamily	Enzyme
229	Q8RDP5	-	Protein of unknown function (DUF2797), Linear amide C-N hydrolases, choloylglycine hydrolase family	-	-	-	-
230	Q8RDT0	-	-	-	-	-	-
231	Q8REV8	-	-	-	-	-	-
232	Q8REV9	-	Domain of unknown function (DUF4397)	-	-	-	-
233	Q8RHF3	-	Transcriptional activator of glycolytic enzyme	-	-	-	-

234	Q8RET4	-	ECF transporter, substrate-specific component, Domain of unknown function (DUF4271), 7th Chemosensory receptor	-	-	-	-
235	Q8RF10	-	Protein of unknown function (DUF1725), Apea-like HEPN, Protein of unknown function DUF86, Tetraspanin family, Jiraiya, SUR7/PalI family, Type II secretory pathway pseudopilin, Transmembrane proteins 230/134, Orf76 (Ac76), Protein of unknown function (DUF3593), Predicted membrane protein (DUF2207), Transmembrane protein 43	-	-	-	-
236	Q8REC4	Abortive infection system protein AbiD/AbiF (Abi_system_AbiD/AbiF), Abortive infection system protein AbiD/AbiF-like (Abi_system_AbiD/AbiF-like)	Abi-like protein, Protein of unknown function (DUF2521)	Abi_2	Abi-like protein (Abi_2)	Abi_2 superfamily	Phage related protein
237	Q8RFA9	DNA alkylation repair enzyme, Armadillo-type fold	DNA alkylation repair enzyme, non-SMC mitotic condensation complex subunit 1	DNA_alkylation	DNA alkylation repair enzyme	AlkD_like superfamily	Enzyme
238	Q8RG02	-	Spo7-like protein	-	-	-	-
239	Q8RHW9	-	Cancer susceptibility candidate 1 N-terminus	-	-	-	-
240	Q8RFQ1	-	-	-	-	-	-
241	Q8RHE1	-	-	-	-	-	-
242	Q8RF54	-	Protein of unknown function (DUF973)	-	-	-	-

243	Q8RGZ1	-	Prefoldin subunit, Exonuclease VII small subunit	-	-	-	-
244	Q8RDZ6	-	Molybdopterin oxidoreductase Fe4S4 domain, Ribosomal protein L37e	-	-	-	-
245	Q8RHB0	-	Dna[CI] antecedent, DciA	-	-	-	-
246	Q8RHP6	-	-	-	-	-	-
247	Q8RH10	DUF4298	Domain of unknown function (DUF4298), Not1 N-terminal domain, CCR4-Not complex component, Exonuclease VII small subunit, Type of WD40 repeatD5	DUF4298	Domain of unknown function DUF4298	DUF4298 superfamily	Function unknown
248	Q8RID5	-	Flavin adenine dinucleotide (FAD)- dependent sulfhydryl oxidase	-	-	-	-
249	Q8RGA5	-	MORN repeat variant, Putative ATP-dependent Lon protease, Lysis protein	-	-	-	-
250	Q8RIP3	-	Protein of unknown function (DUF3417)	-	-	-	-
251	Q8RGV4	-	Poly(A) polymerase central domain, Ribosomal L27e protein family	-	-	-	-
252	Q8RGK6	Protein of unknown function DUF4911	Domain of unknown function (DUF4911), Domain of unknown function (DUF1951)	DUF4911	Domain of unknown function DUF4911	DUF4911	Function unknown
253	Q8RH61	-	SUZ domain	-	-	-	-
254	Q8RI31	-	-	-	-	HDC_Protein superfamily	-
255	Q8RII9	-	Domain of unknown function (DUF4477)	-	-	-	-
256	Q8RI97	-	Protein of unknown function (DUF3781)	-	-	-	-

257	Q8RIE1	-	Domain of unknown function (DUF4234)	-	-	-	-
258	Q8RF09	-	-	-	-	-	-
259	Q8RHP7	-	-	-	-	-	-
260	Q8RE00	-	-	-	-	-	-
261	Q8RGS2	-	-	-	-	-	-
262	Q8REL7	-	Lysine exporter LysO	-	-	-	-
263	Q8RI92	-	PAAD/DAPIN/Pyrin domain, ACT domain	-	-	-	-
264	Q8REX6	-	-	-	-	-	-
265	Q8RHH4	PD-(D/E) XK nuclease superfamily 9 (PDDEXK_9)	PD-(D/E) XK nuclease superfamily, Virulence activator alpha C-term	PDDEXK_9	PD-(D/E) XK nuclease superfamily (PPDEXK_9)	PDDEXK_9 superfamily	Enzyme (restriction endonuclease)
266	Q8RFC5	-	Late competence development protein ComFB	-	-	-	-
267	Q8RGW0	-	-	-	-	-	-
268	Q8RE40	-	-	-	-	-	-
269	Q8RIC0	-	-	No result because of too short sequence	-	-	-
270	Q8RFS7	-	-	-	-	DUF2428 superfamily	-
271	Q8REL5	-	Bacterial domain of unknown function (DUF1798)	-	-	-	-
272	Q8RFJ9	-	-	-	-	-	-
273	Q8REZ1	-	Domain of unknown function (DUF6305), Putative prokaryotic signal transducing protein, LD-Carboxypeptidase N-terminal domain, Domain of unknown function (DUF1967), Ribosomal protein L14	-	Domain of unknown function DUF6305	-	-

274	Q8REL8	-	Nsp 1-like C-terminal region, Tektin family, Initiation control protein YabA, Outer membrane efflux protein, Flagella accessory protein C (FlaC), Short coiled-coil protein, THUMP domain-like, Nucleopolyhedrovirus P10 Protein, emp24/gp25L/p24 family/GOLD, Tetramerisaion domain of TRPM, Takusan, Apg6 coiled-coil region, RNA pol II promoter Fmp27 protein domain, Junction-mediating and -regulatory protein, Gammaherpesvirus protein of unknown function (DUF848)	-	-	-	-
275	Q8RGC3	Protein of unknown function DUF2194, Glycoside hydrolase/deacetylase, beta/alpha-barrel	Uncharacterized protein conserved in bacteria, Polysaccharide deacetylase	DUF2194	Uncharacterised protein conserved in bacteria DUF2194	COG4878 superfamily	Unknown function
276	Q8RES6	-	-	-	-	-	-
277	Q8RGV8	-	-	-	-	-	-
278	Q8RF79	-	Helix-turn-helix domain	-	-	-	-
279	Q8REE9	Septum formation initiator FtsL/DivIC	Septum formation initiator, Domain of unknown function (DUF4349), Autophagy protein ATG17-like domain, Protein of unknown function (DUF3450), Protein of unknown function (DUF1664), Ead/Ea22-like protein, Flagellar Assembly protein A, ESCRT-I subunit Mvb12	DivIC	Septum formation initiator (DivIC)	DivIC superfamily	Cell-division regulatory protein

280	Q8RFG7	-	DEK C terminal domain, Apg6 coiled-coil region, Protein of unknown function (DUF2570)	-	-	-	-
281	Q8RF46	Protein of unknown function DUF4846	Domain of unknown function (4846), YjzC-like Protein	DUF4846	Domain of unknown function DUF4846	DUF4846 Superfamily	Function unknown
282	Q8RFV6	-	-	-	-	-	-
283	Q8RFI6	-	Protein of unknown function (DUF3741), Protein of unknown function (DUF1635), Cop9 signalosome subunit 5 C-terminal domains	-	-	-	-
284	Q8RG54	-	-	-	-	-	-
285	Q8RFL2	-	Hydrogenase/urease nickel incorporation, metallochaperone, hypA, C1 domain, Transposase zinc-ribbon domain, zinc-ribbons, Double zinc ribbon, Phorbol esters/diacylglycerol binding domain (C1 domain), SprT-like zinc ribbon domain, CHY zinc finger, Ubiquitin-binding zinc finger, Zinc-finger of acetyl-transferase ESCO	-	-	HypA superfamily	-
286	Q8RE95	-	-	-	-	-	-
287	Q8RIQ4	-	PF06271, RDD family	-	-	-	-
288	Q8RIL7	Trigger factor/SurA domain superfamily	Family of unknown function (DUF5442)	-	-	-	-
289	Q8RFW7	-	BsuBI/PstI restriction endonuclease domain, Domain of unknown function (DUF4263)	-	-	-	-

290	Q8RFG4	Tautomerase/MIF superfamily, DUF1904	Domain of unknown function (DUF1904), Cytidylate kinase-like family, Izumo sperm-egg fusion, Ig domain-associated, Secreted novel AID/APOBEC-like Deaminase 1, Malarial early transcribed membrane protein (ETRAMP), Carboxylesterase family	DUF1904	Domain of unknown function DUF1904	DUF1904 superfamily	Function unknown
291	Q8RHV3	-	CCR4-NOT transcription complex subunit 1 TTP binding domain, Cut8, nuclear proteasome tether protein, Protein of unknown function (DUF3801)	-	-	-	-
292	Q8REB2	RloB-like protein (RloB)	RloB-like protein, Ubiquitin-2 like Rad60 SUMO-like, Protein of unknown function (DUF1697), Josephin, WGR domain	RloB	RloB-like protein (RloB)	RloB superfamily	Phage related Protein
293	Q8RH59	-	-	-	-	-	-
294	Q8REP2	-	DpnD/PcfM-like protein, PIG-P	-	-	-	-
295	Q8RFJ2	-	-	-	-	HDC_Protein superfamily	-
296	Q8REB3	-	Family of unknown function (DUF6037)	-	Family of unknown function DUF6037	-	-
297	Q8RI49	-	Prokaryotic membrane lipoprotein lipid attachment site, Glycosyl hydrolase family 46, Protein of unknown function (DUF3139)	-	-	-	-

298	Q8RI95	Papain-like cysteine peptidase superfamily, Permuted papain-like amidase enzyme, YaeF/YiiX, C92 Family	Permuted papain-like amidase enzyme, YaeF/YiiX, C92 family, CHAP domain, Bacteriophage peptidoglycan hydrolase, Prokaryotic membrane lipoprotein lipid attachment site	Peptidase_C92	Permuted papain-like amidase enzyme, YaeF/YiiX, C92 family	NLPC_P60 Superfami ly	Enzyme
299	Q8RER1	Flavoprotein-like_sf	NADPH-dependent FMN reductase, Flavodoxin-like fold	-	-	FMN_red superfamil y	Enzyme
300	Q8RGC4	Protein of unknown function DUF2194, Class_I_glutam ine amidotransferas e-like (Class_I_gatase -like)	Uncharacterised protein conserved in bacteria (DUF2194), Family of unknown function (DUF6263), Duffy binding domain	DUF2194	Uncharacteriz ed Protein conserved in bacteriaDU F2194	COG4878 superfmail y	Unknown function
301	Q8RF59	-	Biofilm formation regulator YbaJ	-	-	-	-
302	Q8REJ6	-	Thioredoxin, Thioredoxin-like domain	Thioredoxin_9	Thioredoxin_9	Thioredoxin _like superfamil y	Enzyme
303	Q8REX8	-	-	-	-	-	-
304	Q8RGB9	Outer Membrane Protein/Outerm embrane enzyme PagP, beta-barrel	Outer membrane protein beta-barrel domain	-	-	OM_Channe ls superfamil y	Membrane protein
305	Q8RE01	-	-	-	-	-	-
306	Q8RIC3	-	-	-	-	-	-
307	Q8RF19	-	Cathepsin propeptide inhibitor domain (I29)	-	-	-	-
308	Q8RI48	-	-	-	-	-	-

309	Q8RHE0	-	Glycogen debranching enzyme C-terminal domain, RNA polymerase Rpb1, domain 2, Domain of unknown function (DUF5074)	-	-	-	-
310	Q8RF83	Tetratricopeptide-like helical domain superfamily	Tetratricopeptide repeat, Translation initiation factor IF-2, N-terminal region	TPR_6	Tetratricopeptide repeat	-	Binding protein
311	Q8RE50	-	Sld3 N-terminal domain	-	-	-	-
312	Q8RGV9	-	-	-	-	-	-
313	Q8RDP4	-	Betacoronavirus-like spike glycoprotein S1, N-terminal	-	-	-	-
314	Q8RHL3	GDYXXLXY	GDYXXLXY protein	GDYXXLXY	GDYXXLXY protein	GDYXXLXY superfamily	Function unknown
315	Q8RH94	YfbM-like superfamily	Domain of unknown function (DUF1877)	-	-	DUF1877	Function unknown
316	Q8RGW2	-	-	-	-	-	-
317	Q8RFW6	-	Interferon-induced transmembrane Protein	-	-	-	-
318	Q8RFK7	-	Mga helix-turn-helix domain, Cathepsin propeptide inhibitor domain (I29), Kinetoplastid membrane protein 11	-	-	-	-
319	Q8RDN2	-	-	-	-	-	-
320	Q8RHW2	-	Hydroxymethylglutaryl-coenzyme A reductase	-	-	-	-
321	Q8RE27	-	-	-	-	-	-
322	Q8RIK7	-	-	-	-	-	-
323	Q8RFM6	-	PAS fold	-	-	-	-
324	Q8RG60	-	-	-	-	-	-
325	Q8RE17	-	-	-	-	-	-
326	Q8RFH0	Protein of unknown function DUF4241	Protein of unknown function (DUF4241)	DUF4241	Protein of unknown functionDUF4241	DUF4241	Function unknown

327	Q8REM3	-	Hydroxymethylglutaryl-coenzyme A synthase N terminal	-	-	-	-
328	Q8RIB4	-	-	-	-	-	-
329	Q8RFE3	Winged helix DNA-binding domain superfamily, Winged Helix-like DNA-binding domain superfamily	Bacterial regulatory protein, arsR family, Helix-turn-helix domain, Nuclear RNA-splicing-associated protein, Sugar-specific transcriptional regulator TrmB, Helix-turn-Helix domain, tRNA methyltransferase 5 N-terminal domain, Uncharacterized protein conserved in archaea (DUF2250), Winged helix-turn-helix DNA-binding, ParA helix turn helix domain	-	-	pheS	-
330	Q8RDX6	-	Siphovirus Gp157, Bacteriochlorophyll A protein	-	-	-	-
331	Q8REK3	-	Uncharacterised protein conserved in bacteria (DUF2316)	-	-	PTZ00341 superfamily	-
332	Q8RGY3	-	-	-	-	-	-
333	Q8RH29	Ethanolamine utilization, putative (Eut_put)	Domain of unknown function (DUF6429), CoA binding domain, Hydantoinase/oxoprolinase N-terminal region	-	-	Eut_hyp superfamily	-
334	Q8RG37	-	Xrn1 helical domain, Family of unknown function (DUF5905)	-	-	-	-
335	Q8RH93	-	Protein of unknown function (DUF3269)	-	-	-	-
336	Q8REV5	-	-	-	-	-	-
337	Q8RFB9	-	Protein of unknown function (DUF3763)	-	-	-	-
338	Q8REP9	-	-	-	-	-	-
339	Q8RGQ6	Glycoside hydrolase superfamily	-	-	-	-	-

340	Q8REQ5	-	-	-	-	-	-
341	Q8RHN1	-	-	-	-	-	-
342	Q8RHE8	-	rRNA biogenesis protein RRP36, MinK-related peptide, potassium channel accessory sub-unit protein 4	-	-	-	-
343	Q8REI4	Cysteine protease Prp superfamily, Cysteine protease Prp	Cysteine protease Prp, Carboxypeptidase Y pro-peptide	Peptidase_Prp	Cysteine protease-Prp	Prp-like	Enzyme
344	Q8REN7	-	NADH dehydrogenase transmembrane subunit	-	-	-	-
345	Q8RGA1	-	Polyribonucleotide nucleotidyltransferase, RNA binding	-	-	-	-
346	Q8RG08	-	Malonate/sodium symporter MadM subunit, UbiA prenyltransferase family, NADH-ubiquinone oxidoreductase chain 4, amino terminus	-	-	-	-
347	Q8RFA5	Protein of unknown function DUF4299	Domain of unknown function (DUF4299), Protein of unknown function (DUF1137)	DUF4299	Domain of unknown function DUF4299	DUF4299	Function unknown
348	Q8RG74	-	Chlamydia CHLPS protein (DUF818), Jiraiya	-	-	-	-
349	Q8REC6	CRISPR-associated protein Csx8	CRISPR-associated protein Csx8 (Cas_Csx8), Protein of unknown function (DUF1572)	Cas_Csx8	CRISPR-associated protein Csx8 (Cas_Csx8)	Cas8a1_I-A superfamily	Defense (Function unknown)
350	Q8REX1	-	-	-	-	-	-
351	Q8RGC5	-	-	-	-	-	-
352	Q8RI91	-	-	-	-	-	-

353	Q8RF75	-	TrbC/VIRB2 pilin, SVM protein signal sequence, Small integral membrane protein 3, Predicted membrane protein (DUF2335)	-	-	-	-
354	Q8RH91	-	Domain of unknown function (DUF4908)	-	-	-	-
355	Q8RGY1	-	-	-	-	-	-
356	Q8RGH9	-	-	-	-	-	-
357	Q8RHH0	-	-	-	-	-	-
358	Q8REW6	-	ATP synthase	-	-	-	-
359	Q8RH76	-	-	-	-	-	-
360	Q8RH01	-	-	-	-	-	-
361	Q8RHR5	-	Malarial early transcribed membrane protein (ETRAMP)	-	-	-	-
362	Q8RH12	ABC/ECF transporter, Transmembrane Component	Cobalt transport protein	CbiQ	Cobalt transport protein (CbiQ)	EcfT superfamily	Transport protein
363	Q8RGE8	-	Chordopoxvirus G3 protein	-	-	-	-
364	Q8RE96	Protein of unknown function DUF445	Protein of unknown function (DUF445), Domain of unknown function (DUF5095)	DUF445	Protein of unknown function DUF445	YheB superfamily	Function unknown
365	Q8RGY4	-	-	No result due to short sequence	-	-	-
366	Q8RFW4	-	-	-	-	-	-
367	Q8RIH9	-	Asp23 family, cell envelope-related function, Family of unknown function (DUF6260), Golgi 4-transmembrane spanning transporter	-	-	Anchor_AmaP superfamily	-
368	Q8RI40	-	Aldos-2-ulose dehydratase/isomerase (AUDH) Cupin domain, Protein of unknown function (DUF3641)				-

369	Q8RFK8	-	Transposase DDE domain, Phosphtidylinositol-specific phospholipase C, Y domain	-	-	-	-
370	Q8RHC5	-	-	-	-	-	-
371	Q8RIC7	-	-	-	-	-	-
372	Q8RIM4	Protein of unknown function DUF5706	Family of unknown function (DUF5706), SAVED-fused 2TM effector domain, Protein of unknown function (DUF1218)	-	Family of unknown function DUF5706	DUF5706 superfamil y	Function unknown
373	Q8RG38	-	Domain of unknown function (DUF4077), Transglutaminase elicitor	-	-	PLN0246 superfamil y	-
374	Q8RH73	Protein of unknown function DUF3290	Protein of unknown function (DUF3290), Domain of unknown function (DUF4579), 7TM diverse intracellular signaling, Family of unknown function (DUF6341), Protein of unknown function (DUF1689), Protein of unknown function (DUF2678), Domain of unknown function (DUF4131)	DUF3290	Protein of unknown function DUF3290	DUF3290 superfamil y	Function unknown
375	Q8RF03	-	7TM diverse intracellular signaling, Major facilitator superfamily, Positive regulator of sigma(E), RseC/MucC, Replication factor RFC1 C terminal domain, Domain of unknown function (DUF373), 2TM domain	-	-	-	-
376	Q8RHP8	-	-	-	-	-	-
377	Q8RGG0	Positive Regulator of Sigma(E), RseC/MucC	Positive regulator of sigma(E), RseC/MucC	RseC/MucC	Positive regulator of sigma(E), RseC/MucC	RseC_MucC superfamil y	Regulatory protein
378	Q8RHC3	-	-	-	-	-	-
379	Q8RFH7	-	-	-	-	-	-

380	Q8RHV9	-	Desulfoferrodoxin, N-terminal domain	-	-	-	-
381	Q8RH69	Protein of unknown function DUF896	Bacterial protein of unknown function (DUF896)	DUF896	Bacterial protein of unknown function (DUF896)	DUF896 superfamily	Function unknown
382	Q8RGP0	-	-	-	-	-	-
383	Q8RGP7	-	Integral membrane protein (DUF2244)	-	-	-	-
384	Q8RDV5	-	-	-	-	-	-
385	Q8RE05	-	Short chain fatty acid transporter	-	-	-	-
386	Q8RGX9	-	-	-	-	-	-
387	Q8RGQ2	-	Male sterility protein	-	-	-	-
388	Q8RF70	Protein of unknown function DUF4125	Protein of unknown function (DUF4125)	DUF4125	Protein of unknown function (DUF4125)	DUF4125 superfamily	Function unknown
389	Q8RI41	Protein of unknown function DUF1266	Protein of unknown function (DUF1266)	DUF1266	Protein of unknown function DUF1266	DUF1266 superfamily	Function unknown
390	Q8RDW0	-	-	-	-	-	-
391	Q8RF29	Transcription Regulator Rrf2, Winged Helix-like DNA-binding domain superfamily, Winged Helix DNA-binding domain superfamily	Iron-dependent Transcriptional regulator, Winged helix-turn-helix DNA-binding, Sugar-specific transcriptional regulator TrmB, LlaMI restriction endonuclease	Rrf2	Iron-dependent Transcriptional regulator (Rrf2)	HTH superfamily	Regulatory protein
392	Q8RIA8	-	-	-	-	-	-
393	Q8R6H9	-	Uncharacterized protein PXO2-72	-	-	-	-

394	Q8R6K0	Adhesion Protein FadA	Adhesion protein FadA, SWI5-dependent HO expression protein 3, RecC C-terminal domain, HEPN/Toprim N-terminal domain 1, Plasmodium variant antigen protein Cir/Yir/Bir, Coiled-coil domain-containing protein 23, Protein of unknown function (DUF812), Glycosyl hydrolase family 65 central catalytic domain, Flagellar assembly protein A, Fibrinogen alpha/beta chain family, Uncharacterised protein family (UPF0242) N-terminus	FadA	Adhesion protein FadA	FadA superfamil y	Other
395	Q8R6I0	-	-	No result due to short sequence	-	-	-
396	Q8R6K1	-	-	-	-	-	-
397	Q8RHM0	-	Domain of unknown function (DUF5604), Archease protein family (MTH1598/TM1083)	-	-	-	-
398	Q8RHF0	-	-	-	-	-	-
