# Supplementary material for: Functional annotation of uncharacterized proteins from Fusobacterium nucleatum: identification of virulence factors
Source: Genomics Inform. 2023 Jun 30;21(2):e21. doi: 10.5808/gi.22065 (PMC10326533; doi:10.5808/gi.22065)
Supplement: Supplementary Table 6. — Protein-protein interaction of uncharacterized proteins from Fusobacterium nucleatum ATCC 25586 [file gi-22065-Supplementary-Table-6.pdf]

Supplementary Table 6. Protein-protein interaction of uncharacterized proteins from *Fusobacterium nucleatum* ATCC 25586

S. No.	Accession ID	Neighborhood	Gene fusion	Co-occurrence	Co-expression	Experiments	Databases	Text mining	Homology	Confidence score
1	Q8RDP1	8	0	7	0	0	0	5	0	2.5
2	Q8RDY5	1	0	10	0	0	0	0	0	1.375
3	Q8REC7	8	0	8	2	1	0	4	0	2.875
4	Q8REG3	3	2	2	0	0	0	1	0	1
5	Q8REM4	3	0	9	0	2	0	0	0	1.75
6	Q8RER4	7	0	7	5	0	0	2	0	2.625
7	Q8RFD4	1	0	9	0	4	0	0	0	1.75
8	Q8RFF3	10	0	1	1	0	0	0	0	1.5
9	Q8RFU1	10	1	8	0	0	0	5	0	3
10	Q8RGC0	10	0	6	3	2	0	3	0	3
11	Q8RGQ9	8	2	4	1	0	0	1	0	2
12	Q8RHE9	9	0	0	4	2	0	5	0	2.5
13	Q8RHQ2	8	3	1	8	4	5	0	0	3.625
14	Q8RHR3	5	1	6	0	0	0	0	0	1.5
15	Q8RHS6	7	2	4	0	0	0	0	0	1.625
16	Q8RII7	10	0	0	0	0	0	0	0	1.23
17	Q8RE80	6	0	10	3	0	0	4	0	2.875
18	Q8REK7	6	0	0	1	0	0	3	0	1.428
19	Q8REQ3	1	0	10	0	0	0	0	0	1.25
20	Q8RF13	10	0	1	3	0	0	0	0	1.75
21	Q8REC4	4	0	9	0	0	0	0	0	1.625
22	Q8RHH4	4	2	8	0	0	0	0	0	1.75
23	Q8REE9	10	0	0	2	3	0	4	0	2.375
24	Q8REB2	6	0	7	0	0	0	0	0	1.625
25	Q8RER1	5	1	7	2	0	0	3	0	2.25
26	Q8REJ6	5	0	5	3	3	2	3	0	2.625
27	Q8RF83	2	0	10	0	0	0	0	1	1.625
28	Q8REI4	6	0	2	0	0	0	3	0	1.375
29	Q8REC6	8	0	0	0	0	0	0	0	1
30	Q8RF29	7	0	0	1	0	0	3	0	1.375
