# Supplementary material for: Functional annotation of uncharacterized proteins from Fusobacterium nucleatum: identification of virulence factors
Source: Genomics Inform. 2023 Jun 30;21(2):e21. doi: 10.5808/gi.22065 (PMC10326533; doi:10.5808/gi.22065)
Supplement: Supplementary Fig. 1. — Structural modeling. Homology based structure prediction and Ramachandran analysis of Q8RFU1 (A), Q8RGP8 (B), Q8RHS6 (C), Q8REI4 (D), Q8RFD4 (E), and Q8RG23 (F). [file gi-22065-Supplementary-Fig-1.pdf]

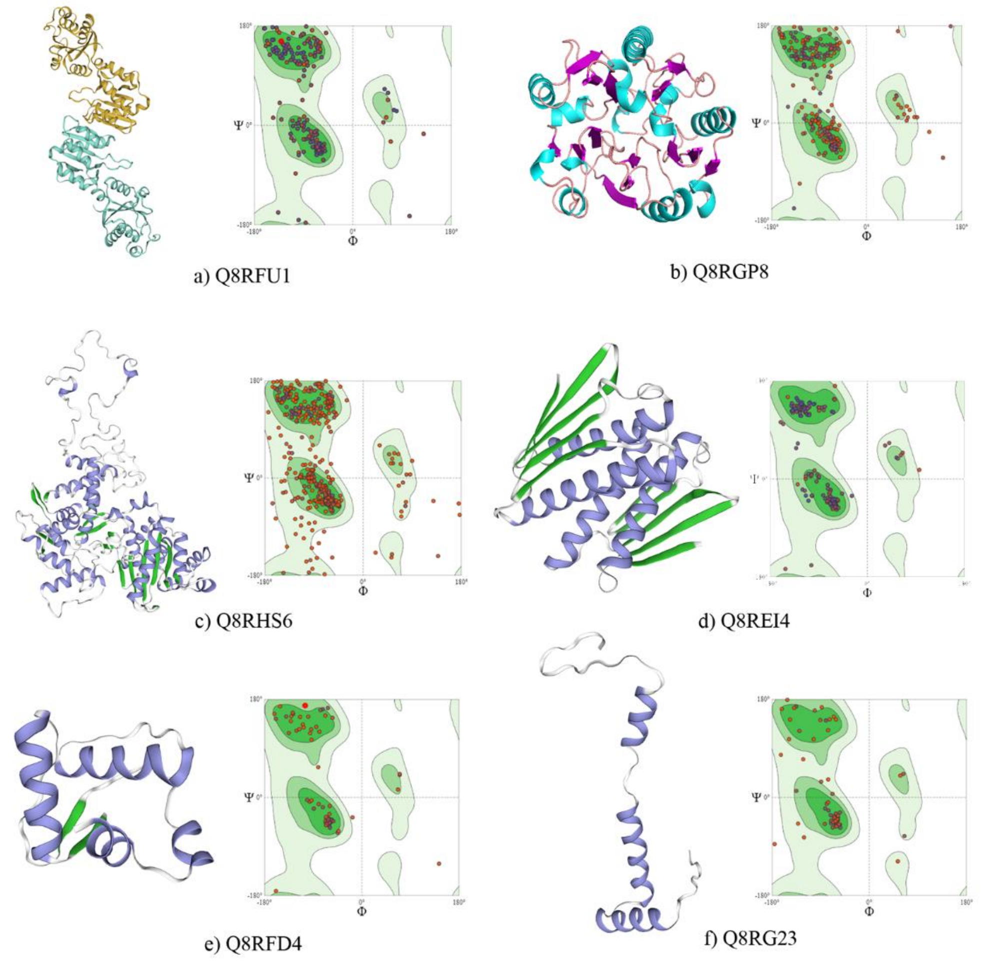

Supplementary Fig. 1. Structural modelling. Homology based structure prediction and Ramachandran analysis of Q8RFU1 (A), Q8RGP8 (B), Q8RHS6 (C), Q8REI4 (D), Q8RFD4 (E), and Q8RG23 (F).
